# Landscape of Herbal Food Supplements: Where Do We Stand with Health Claims?

**DOI:** 10.3390/nu17091571

**Published:** 2025-05-02

**Authors:** Slađana Vojvodić, Dunja Kobiljski, Branislava Srđenović Čonić, Ljilja Torović

**Affiliations:** 1 Department of Pharmacy, Faculty of Medicine, University of Novi Sad, Hajduk Veljkova 3, 21000 Novi Sad, Serbia; sladja.vojvodic@uns.ac.rs (S.V.); branislava.srdjenovic-conic@mf.uns.ac.rs (B.S.Č.); 2 Department of Industrial Engineering and Management, Faculty of Technical Sciences, University of Novi Sad, Trg Dositeja Obradovića 6, 21000 Novi Sad, Serbia; dunjakobiljski@uns.ac.rs; 3 Center for Medical and Pharmaceutical Investigations and Quality Control, Faculty of Medicine, University of Novi Sad, Hajduk Veljkova 3, 21000 Novi Sad, Serbia

**Keywords:** communication, consumer, database, labelling, market transparency, public health

## Abstract

**Background/Objectives**: Health and nutrition are increasingly important to people, which has increased the popularity of products promoted for their contribution to health, such as food supplements. **Methods**: This study encompassed 87 herbal food supplements, assessing the compliance of health claims with regulatory requirements. The study was conducted in Serbia, a European country with a regulatory framework harmonized with the one in the EU; however, it requires the pre-market registration of supplements. **Results**: Health claims were listed on as many as 86.2% of the labels, but only 10.7% of them, all associated with vitamin and mineral ingredients, were compliant with the EU Register of authorized health claims. An additional 38.7% of supplements carried “on-hold” claims from the EFSA Register of questions for botanicals. The remaining ones (50.6%) comprised those attributed with strictly prohibited properties of disease prevention, treatment, or cure (9.3%), and those containing at least one botanical-related health claim out of the scope of the Register of questions. **Conclusions**: The study unequivocally showed the worrying lack of adherence to regulations in the Serbian settings. Considering the importance of labelling for consumer protection and public health, the authors of this paper advocate for significant improvement in quality assurance of the registration process, tightening of market control, and an effective solution for on-hold claims on botanicals continuously used under transitional regulatory measures.

## 1. Introduction

In today’s society, the awareness of the importance of healthy lifestyles, with healthy nutrition being the first among the keys to a long and quality life [1], is on the rise. An optimal, well-balanced diet is no longer considered sufficient; non-nutritive biologically active compounds promise benefits beyond a satisfactory health status (“normal function”). There is an evident expansion of the intake of herbal food supplements, produced from a huge range of plants having an army of constituents with potentially beneficial effects on health [2]. And there is an important question: How can consumers be informed about the health benefits? A simple, yet potentially extremely effective tool is the use of health claims on supplement labels and advertisements. Given their potential to influence consumer choice, it is important to investigate the regulatory compliance of voluntary health claims made on herbal food supplements.

The estimated value of the worldwide supplements market stood at USD 177.50 billion in 2023, with a projected Annual Growth Rate of 9.1% from 2024 to 2030 [3]. Findings of the European Union (EU) consumer survey showed that 88% of respondents reported having used a food supplement at some stage in their lives, with the overwhelming majority of this group (93%) within the past 12 months [4]. The consumption of food supplements is increasing globally, primarily due to their availability and the fact that complementary and alternative therapies are being highly accepted. Such acceptance led to the increased use of herbal products, due to dissatisfaction with conventional drugs and consumers’ perception that all natural (herbal) products are healthy and do not pose any risk for human health [5,6]. Moreover, in light of the COVID-19 pandemic, there has been heightened interest in alternative approaches to conventional medicines for boosting immunity and reducing the risk of infection [7,8,9,10].

### 1.1. Regulatory Framework for Food Supplements

In the EU, food supplements represent concentrated sources of nutrients (vitamins, minerals) or other substances with nutritional or physiological effect (amino acids, enzymes, prebiotics, probiotics, essential fatty acids, fibre, herbal extracts, etc.), available in dosage forms to supplement the normal diet [11]. Food supplements fall under the general food law [12], more specifically Directive 2002/46/EC [11]. There is no centralized pre-market authorization [13], although some EU member states require a note of authorization to place a supplement on the market [7]. According to Regulation 2015/2283 on novel foods [14], only if a product intended to be used in food supplements does not have a history of safe use in the EU before 1997, a new production process has been applied, or it contains nanomaterials, it is classified as a novel food, which requires a safety assessment by the European Food Safety Authority (EFSA). Pre-market efficacy and safety assessment of food supplements is not in the provisions of the EU legislation. However, the belief of most consumers is exactly the opposite, which, combined with the expectation that manufacturers are obliged to inform consumers of known adverse effects [15], could be quite challenging. A centralized nutrivigilance system for the notification of adverse effects associated with food supplements does not exist. However, some operating systems could be used for warning related to food supplements, such as the International Nutrivigilance network (adverse effects), the Rapid Alert System for Feed and Food (RASFF; risks related to composition, contamination, and some labelling issues), and the Emerging Risks Exchange Network (EREN) [16].

### 1.2. Consumer Information Regulatory Framework

An extremely rapid increase in the number and versatility of health claims voluntarily presented on food supplements labels has been evident for years. The products’ labelling should enable consumers to identify and choose appropriate products which meet their individual (nutritional) needs [2]. In the EU, food information to consumers is specified by Regulation 1169/2011 [17], while specific rules regarding the voluntary use of nutrition and health claims are defined by Regulation 1924/2006 [18,19] (Table 1). In general, health claims shall be scientifically substantiated and truthful, based on scientific evidence that supports the association between the intake of a specific ingredient and health benefits. Appropriate warning statements are mandatory as opposed to the voluntary use of health claims. Usage limitations and special warnings for potentially vulnerable categories such as children, pregnant and lactating women, or people suffering from certain chronic diseases shall be stated.

As noted in the European Commission report evaluating Regulation 1924/2006 concerning health claims made on plants and their preparations, issued in 2020 [20], “no health claim on plant substances used in foods received a favorable assessment by EFSA, mainly due to the absence of human intervention studies”. Consequent suspension of the authorization procedure led to an “on-hold” list of 2078 health claims related to plant substances (Regulation 432/2012) [21]. These claims may still be used under the responsibility of the business operators, provided that they comply with Regulation 1924/2006, while pending a final decision [22].

### 1.3. Existing Knowledge and Research Gap

The volume of the published research on food supplement labelling/advertising related to claims and statements follows the sharp increase in the number and versatility of available supplements on one hand and professional and public (consumer) interest on the other. The studies investigating supplement labelling regulatory adherence in different countries indicate that, even where explicit regulations mandating the labelling of supplements are in place, a significant share of products still fails to meet the standards, bearing misleading claims [22]. It should be kept in mind that food information regulations cover not only labels on products themselves (example studies investigating physical labels are studies from Serbia [23,24], Slovenia [25], and USA [26,27]), but also various means of marketing, such as newspaper and TV advertising (example studies investigating magazine, radio and TV commercials are studies from Poland [28], Spain [29], and USA [30]), as well as internet marketing (example studies [16,31,32,33]), which is increasingly represented today, all of them revealing marketing practices that are used to circumvent the regulations.

Along with market availability and affordability, claims/statements can be a powerful tool guiding consumers’ food purchase decisions. Very high consumption rates [4] underline the importance of informed decision-making regarding whether and which supplements to use. However, it is questionable whether consumers know/care and whether they should believe/appreciate what they are told. Regarding supplements based on botanicals, the “unknowns” are vast, as reflected in the provisional status of on-hold claims vs. authorized claims for active components other than botanicals. However, such supplements are rarely the subject of research (a study investigated 13 common herbal products available at websites accessed via the Google search engine [32]), leaving an immense gap in the existing knowledge.

This study is a case study of a European country with a regulatory framework harmonized with that applied in the EU. However, unlike the EU, Serbian law requires an official pre-market registration of food supplements [34,35]. The evaluation of the landscape of herbal supplements available on the market, specifically the spectrum of health claims, precautionary and warning statements presented on their labels, in terms of regulatory compliance, should exemplify how regulations are adhered to in one specific setting, such as in Serbia. The study aims to investigate how supplement producers engage with the current regulatory landscape—do they adhere to it, circumvent it, or overstep it? And do the authorities responsible for mandatory registration effectively hinder the presence of noncompliant supplements on the market? The answers to these questions can contribute to health-related communication between all interested parties—supplement producers, consumers, health professionals, and authorities.

## 2. Materials and Methods

### 2.1. Herbal Food Supplement Collection

The Serbian Ministry of Health maintains a database of food supplements, based on the data obtained through the mandatory pre-market registration, with obligatory registration renewal every five years. The database was established in 2014; however, its format does not allow automatic searching, and publicly available data have not been regularly updated. Hence, to provide access to information presented on supplement labels, food supplements containing herbal extracts or propolis formulated as liquids, syrups, oral sprays, and drops, were purchased in pharmacies, drugstores, and “health food” stores located mainly in the city of Novi Sad (the second largest city in Serbia), and transported to the laboratory. The final collection of supplements marketed in 2021–2023 (various domestic and imported brands) accounted for 87 products, in randomly taken single original unit packages. The number of supplement samples was limited by their commercial availability to the consumers. All relevant data from all the labels were rewritten into an Excel spreadsheet (Microsoft Excel, v2021) (presented in the Supplementary Materials, Table S1) and used for further data processing. All sides of the packages were photographed for the laboratory archive.

### 2.2. Evaluation of Regulatory Compliance of Health Claims/Statements

Health claims from supplements’ labels were paired with relevant claims from open-access databases, i.e., the EU Register of health claims, or, in case of botanicals, the EFSA list of questions [19,21], thus facilitating compliance assessment. The compliance of mandatory, warning, and precautionary statements was assessed against the regulatory requirements specified in Table 1 [11,17,18].

The regulatory compliance of the health claims/statements was assessed through independent evaluation by two reviewers with extensive professional experience and a discussion to reach a consensus on the compliance, considering the following criteria:

- Whether the health claims were following the EU Register of authorized health claims and “on-hold” claims for herbal food supplements from the EFSA Register of questions [19,21]
- Whether the health claims attribute the properties of prevention, treatment, or cure of diseases, which is not allowed according to Directive 2002/46/EC [11] and Regulation 1169/2017 [17].
- Whether the labels included the mandatory statements related to the importance of varied diet and a healthy lifestyle and compliance with recommended doses, as well as necessary precautionary and warning statements which shall follow the health claims on the labels according to Directive 2002/46/EC [11] and Regulation 1924/2006 [18].

According to a previously agreed-upon operational criterion, a supplement bearing multiple health claims was classified as “partially non-compliant” if some of the associated claims matched either “on-hold” or authorized claims, and some did not. The percent agreement calculation of inter-rater reliability before consensus revealed a satisfactory initial level of agreement between the two raters, set at a minimum of 75% (achieved > 80%). The remaining assessments were resolved through a discussion held to reach consensus, and only in indecisive cases (not necessarily because of disagreement, in some cases because of uncertainty of both raters), the raters consulted an independent expert.

After the compliance assessment, the supplements were further sorted, depending on the type of health claims presented (no claims; claims for substances other than botanicals/botanicals/both) and compliance status (fully compliant/non-compliant/“partially non-compliant”), and counted (Supplementary Materials, Tables S2–S7—health claims; Table S8—mandatory, warning, and precautionary statements).

## 3. Results

### 3.1. Regulatory Compliance of Health Claims

**Figure 1:**
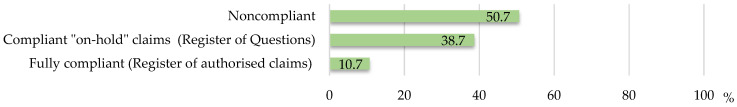
The distribution of herbal food supplements bearing health claims according to their regulatory compliance.

Of the 87 evaluated herbal food supplements, 12 (13.8%) had no health claims on their labels (Table S2). The full spectrum of health claims provided on the labels of the remaining 75 products is presented in Tables S3–S7, while Table 2 presents representative examples. The distribution of the products bearing health claims according to their regulatory compliance is shown in Figure 1.

Out of 75 products with listed health claims, only eight were fully compliant with the legislation (example N1 in Table 2 and Table S3). However, ingredients that carried the claims compliant with the Register of authorized claims were not botanicals. Those were vitamins (vitamins C, D, E, A, B5, B6, B12, and folic acid) and minerals (zinc, copper, iron, calcium, and iodine), accompanying herbal ingredients. Additionally, 29 products carried “on-hold” claims for herbal ingredients compliant with those listed in the EFSA Register of questions (example N2 in Table 2 and Table S4). Among these 29 products, 5, in addition to “on-hold” claims, also had claims from the Register of authorized claims (example N3 in Table 2 and Table S4). The remaining half of the products (38 of them) did not comply with the regulations, i.e., they had claims which were neither in the Register of authorized claims nor on the list of “on-hold” claims. Of these, 21 products listing multiple claims were classified as “partially matching”, as some of the claims presented on the same supplement matched either “on-hold” or authorized health claims, and some did not (example N4 in Table 2 and Table S5). Nine products had claims that did not match (example N5 in Table 2 and Table S6), and eight had claims for ingredients for which there were no defined “on-hold” health claims for the stated purposes (example N6 in Table 2 and Table S7).

It is important to emphasize that the compliance of “on-hold” claims was evaluated concerning their status as approved for use, as defined by the regulation, where such claims are observed as scientifically based and justified, even though they have not yet been assessed by expert bodies. Therefore, after these claims undergo evaluation, their status could become questionable. Moreover, although the authorized and “on-hold” health claims given by the regulation refer to individual supplement ingredients, it was observed that these claims were in some cases attributed to the product as a whole, which is ambiguous and can mislead consumers to the conclusion that only that specific product provides claimed benefits. Misleading claims are prohibited.

**Figure 2:**
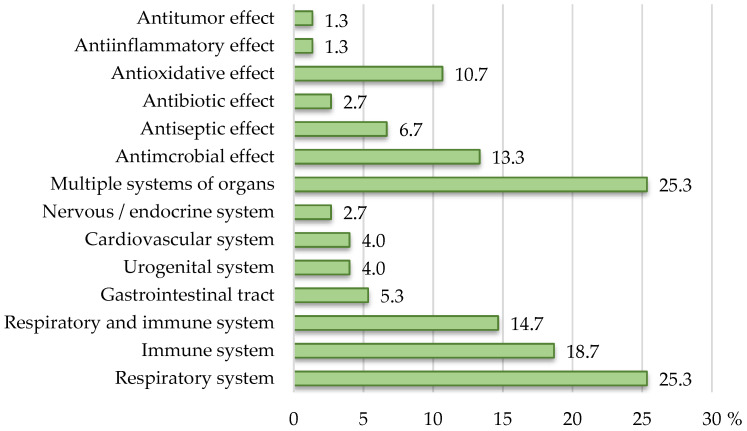
The distribution of herbal food supplements according to the claimed effect on a particular system of organs/specific activity.

The largest number of the products (more than half) carried health claims related to effects on the respiratory and/or immune systems, namely 19 products (25.3%) on the respiratory, 14 (18.7%) on the immune, and 11 (14.7%) on both the respiratory and immune systems. A smaller number of products carried claims about the effects on the gastrointestinal tract (4) and the urogenital (3), the cardiovascular (3), and the nervous systems (2). The effect on several systems of organs was claimed by as many as a quarter of the products (19 out of 75) bearing claims. The distribution of the products according to the claimed effect on a particular system of organs or specific activity is shown in Figure 2.

The most common claims concerning the effect on the respiratory system were carried by herbal ingredients such as primrose, thyme, marshmallow, rosehip, plantago, chamomile, basil, mint, and lemon balm. The largest number of claims stated the contribution to the maintenance of the normal function of the respiratory tract and/or the mucous membranes of the respiratory tract, as well as the purpose of being used by persons with cold and flu symptoms accompanied by a cough, and for the relief of irritation of the mouth and throat mucous membranes. The property of increasing bronchial secretion, reducing secretion density, and facilitating expectoration and breathing by dilating the bronchi has been claimed for primrose and its active ingredients, saponins. In addition to the above, primrose and thyme (and their active ingredient thymol) were attributed expectorant, bronchospasmolytic, and antiseptic effects, while menthol carried the claim of mild local anesthetic effect, ease of breathing, and relief of cold symptoms. Plantago, marshmallow, chamomile, and basil (and their active ingredients mucus, essential oils, saponosides) on the majority of labels were attributed the purpose of maintaining the normal function of the respiratory system’s mucous membranes and helping with inflammation of the respiratory tract, as well as being used for cold and flu symptoms and irritation of the mouth and throat mucous membranes accompanied by a dry cough and heavy expectoration, or were connected with claims such as “for healthy mucous membranes and the health of respiratory organs”. Although there are no defined authorized or “on-hold” health claims for honey, two products claimed that honey could contribute to the health of the respiratory tract due to the presence of phytochemicals with antioxidant properties.

The most important ingredients (herbal raw materials) responsible for the effects on the immune system were acerola, echinacea, elder, beta-glucan, blueberry, rose hip, astragalus, ginseng, and propolis, most of which were claimed to contribute to the normal function of the immune system and the increased resistance of the organism. In addition, there were claims for astragalus, highlighting its contribution to the general physical condition and body protection from external agents.

The effects on the gastrointestinal tract were claimed on the labels of the products containing primarily chamomile, licorice, caraway, lemon balm, mint, and fennel, and to a lesser extent, gentian, common centaury, fenugreek, and alder buckthorn. In addition to contributing to the normal function of the digestive tract, the labels claimed that these herbal raw materials could be used for indigestion, flatulence, gas, and stomach cramps.

Helping with infections of the urinary system, as well as menstrual and menopausal problems, were the main effects claimed by products that contained herbal raw materials with effects on the urogenital tract, such as Lady’s mantle, bearberry (uva), or vitex. Claims for Lady’s mantle present its beneficial effect in urinary infections, inflammation, and polycystic ovaries, as well as in the regulation of the menstrual cycle and comfort before and during the menstrual cycle. Uva was presented as an uro-antiseptic in urinary tract infections, as well as a mild diuretic, while vitex was claimed to improve women’s general condition in the period preceding the menstrual cycle, alleviate menstrual and menopausal problems, and help women with irregular cycles.

Regarding the effect on the cardiovascular system, chestnut, yarrow, and melilot were claimed to maintain physiological blood flow through the venous vessels of the lower extremities and improve the health of venous vessels and capillaries. White mistletoe and hawthorn claimed maintenance of normal heart function and normal cardiac activity. Garlic and wild garlic are claimed to be used for blood circulation disorders and high blood pressure. Garlic was additionally claimed to have antiseptic and antimicrobial effects.

Products based on hops, valerian, mint, lemon balm, thyme, lavender, St John’s wort, and basil claimed to have an effect on the nervous system in the form of a relaxing effect and use for irritability, tension, restlessness, feelings of anxiety or fear, concentration disorders, depression, and stomach disturbances caused by nervousness. Lemon balm carried claims about its contribution to good cognitive function, relaxation, and preservation of normal sleep, while valerian carried claims about its contribution to maintaining mental well-being, and hawthorn about reducing tension and facilitating the onset of sleep.

The products containing valerian, mint, lemon balm, thyme, lavender, St John’s wort, and basil claimed effects on the endocrine system via regulation of hormonal balance. The products containing the Lady’s mantle claimed the regulation of female hormone secretion, while those containing lemon balm claimed preservation of hormonal balance.

Plantago, which contains tannins, pectins, citric acid, vitamin C, saponosides, mucilage, oils, and phytoncides, has been attributed an antibacterial effect. An antimicrobial effect was claimed on the labels of products based on primrose and plantago, with an antiseptic effect claimed for chamomile, thyme (thymol), and menthol. In general, an antimicrobial effect was claimed on labels of ten products (13.3%), an antiseptic effect on five, an antibiotic effect on two, and antioxidant effect on eight, and an anti-inflammatory and antitumor effect on one (Figure 2). Although no authorized or “on-hold” health claims have been defined for propolis, two products claimed that propolis is a natural antibiotic, as well as that it exhibits antibacterial, antifungal, antiviral, antiparasitic, antioxidant, and immune-stimulating effects, as well as having a beneficial effect on strengthening the body’s resistance. It was claimed that it can be used in various inflammations, as well as in gynecology and dermatocosmetology, and in superficial wounds and infections on the skin and mucous membranes, due to its property of stimulating the processes of regeneration and epithelization of damaged tissue. An additional product based on propolis claimed the bacteriostatic, bactericidal, antiviral, antifungal, immune-stimulating, and antitumor properties of propolis.

### 3.2. Attribution of Disease Prevention, Treatment, or Cure Properties

A total of 7 (9.3%) of the 75 examined products presented on their labels at least one of the following terms, “prevention” (4), “treatment” (1), “disease” (1), and “therapy” (1), thus attributing properties of disease prevention, treatment, or cure to the product, which is not allowed [18] and misleads consumers. Additional products listed claims such as “helps with…”, “has (beneficial) effect…”, “to be used in (various health conditions)” such as cough, cold and flu, reducing inflammation, rheumatic pain, hypertension, anxiety, insomnia, regulation of metabolism, hormonal balance, etc., as stated above and as can be seen in detail in Tables S2–S7. Moreover, claims such as “to be used as an expectorant or diuretic” or “has antimicrobial, antiviral, antifungal, antitumor, antiseptic, spasmolytic, anti-inflammatory, antioxidant and/or other effects” or “contributes to the health or normal function of the respiratory/digestive/urinary/immune system…” could mislead an average consumer and be understood as medicinal properties.

### 3.3. Regulatory Compliance of Precautionary and Warning Statements

**Figure 3:**
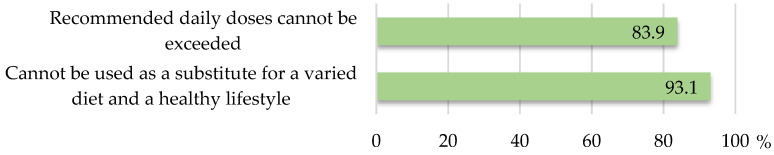
The proportion of herbal food supplements bearing mandatory statements on their labels.

The statement that supplements cannot be used as a substitute for a varied diet and a healthy lifestyle was not found on 6 product labels (6.9%), of which 5 (6.7%) belonged to the group of 75 products with listed health claims. All six highlighted supplements also lacked the mandatory warning that recommended daily doses cannot be exceeded, which was absent from a further eight labels (in total, fourteen, 16.1%). The distribution of the products concerning the presence of mandatory statements is shown in Figure 3.

**Figure 4:**
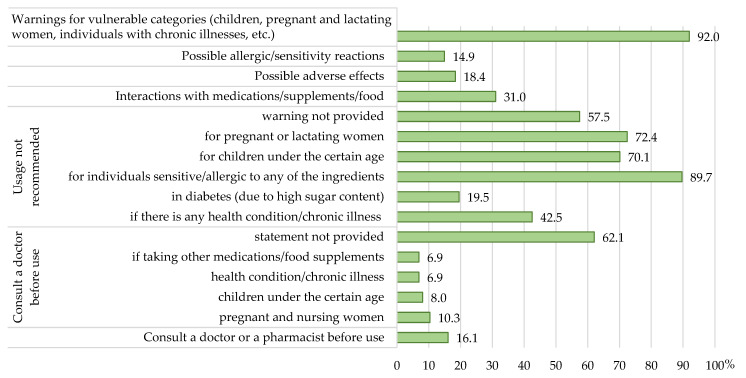
The distribution of herbal food supplements according to the precautionary and warning statements.

Table S8 presents the full spectrum of precautionary and warning statements on the usage of the evaluated herbal food supplements, as taken from their labels; the distribution of the products according to the labelled precautions/warnings is shown in Figure 4.

It was observed that only seven products (8.0%) did not present warnings for vulnerable categories: children, pregnant and lactating women, people suffering from chronic diseases, etc. The warning/precautionary statement that the product is not recommended for use by those hypersensitive/allergic to any of the product’s ingredients was present on 78 labels (89.7%), that the product is not recommended for use by children younger than a certain age (following the specific use of a products for the appropriate age for which was intended for) was present on 61 labels (70.1%), and that the product is not recommended for use by pregnant and lactating women was present on 63 labels (72.4%).

The statement that it is necessary to consult a doctor or a pharmacist before use was present on 14 labels (16.1%), while the statement that it is needed to consult a doctor before use in specific categories was present on 9 products in the case of pregnant and lactating women, 7 in the case of children younger than a certain age, 6 products if there was a medical condition/chronic disease, and 6 products if consumers were taking other medicines/food supplements, while 54 products (62.1%) did not present such statements.

Labels of 37 products (42.5%) stated that the product is not recommended if there is a medical condition/chronic disease. The most frequently referred disease was diabetes (17 out of 37, 46.0%), and warnings were related to the high sugar content of the product. However, as many as 50 products (57.5%) did not present such warnings/precautions, although numerous herbal ingredients of these supplements require warnings related to restrictions in their usage. A warning related to interactions with other supplements/foods/drugs was claimed for 27 products (31.0%), while possible side effects were notified on 16 (18.4%), and allergic reactions (hypersensitivity) on 13 (14.9%).

## 4. Discussion

The studies investigating supplements labelling regulatory adherence in different countries indicate a variable extent of the common problems as revealed in the current research. The main findings concerning different communication channels (printed product labels; magazine, radio, and TV advertising; internet marketing) are given in Table 3.

Comparisons between the studies should be taken with due caution; in the lack of a sufficient number of studies on claims concerning botanicals, to which a specific legal framework is attached (on-hold claims vs. authorized claims for active ingredients other than botanicals), data on very diverse supplements are shown. In comparison with a more than decade old Serbian study [23], conducted on multivitamin and mineral supplements, the current one showed an almost doubled proportion of products presenting health claims (86.2%), while the proportion of those fully compliant with regulations was threefold lower (10.7%). Significant improvement observed in the use of expressions such as “prevention”, “treatment”, or “therapy” (9.3 vs. 25.0%) could be an achievement of the pre-market registration process introduced in 2014. A recent Serbian study [24] on omega-3 fatty acid supplements found that 70.1% of the products featured health claims related to omega-3 fatty acids. However, the proportion of products fully compliant with regulations was significantly higher (86.8%) than in the current study. The authors also analyzed the relationship between the regulatory compliance of a product’s health claims and factors such as country of origin, source of active substance, target population, and price per daily dose. The findings indicate that none of these factors had significant explanatory power for compliance, suggesting that even a high-priced product does not guarantee the accuracy of the health claims. The validity assessment of claims based on omega-3 fatty acids intake from food supplements, in comparison with the intake requirements defined for authorized claims, supported 85% of the claims. Regarding the use of mandatory warnings, both studies yielded similar findings. The statement that food supplements cannot be used as a substitute for a varied diet and a healthy lifestyle appeared in 93.1% of products in the current study versus 86.6% in the omega-3 fatty acid study. Similarly, the mandatory warning that recommended daily doses cannot be exceeded was present in 83.9% vs. 80.4% of the products, respectively. However, differences were observed in the presence of other warning/precautionary statements. The warning that the product is not recommended for individuals hypersensitive or allergic to any of its ingredients was included in 89.7% of the products in the current study, compared to 45.4% in the omega-3 fatty acid study. Likewise, the statement that the product is not recommended for children below a certain age appeared in 70.1% of the products in the current study compared to 35.1% in the omega-3 fatty acid study. These differences most probably reflect a huge diversity of compounds contained in herbal ingredients, as opposed to omega-3 fatty acids, as well as many “unknowns” regarding herbal constituents and their effects on humans. However, the recommendation to consult a doctor or pharmacist before use was present in only 16.1% of the products in the current study vs. 49.5% in the omega-3 fatty acid study. A 2023 U.S. study [26] evaluated the labelling of 51 physical Ayurvedic herbal supplements and the online product descriptions of 42 products purchased online. Among the physical product labels, 60.8% exhibited at least one regulatory noncompliance. Specifically, 33.3% lacked or had noncompliant disclaimers for structure/function or general well-being claims, 29.4% had a missing or noncompliant “Supplement Facts” label, 27.5% displayed a noncompliant statement of identity, and 25.5% lacked a compliant domestic mailing address or phone number. The study highlights that the use of disease claims on herbal supplements poses a significant public health concern, as such claims may lead consumers to delay seeking professional medical treatment. Disease claims were identified in 38.1% of online product descriptions and 7.8% of physical product labels.

A Slovenian study [25], conducted to evaluate the use of mandatory and voluntary food supplements and novel food information, identified various errors on the supplement labels, spanning from typographical errors to misleading practices, also proven in the current study.

A study performed on the content of audio-visual advertisements aired on TV and radio stations in Poland showed that almost one third (28.2%) of the promoted supplements made unsubstantiated claims regarding their effectiveness [28], which is around two-fold lower than the percentage of noncompliant supplements (50.7%) recorded in the current study.

The current era of internet marketing and the expansion of online selling pose novel regulatory hurdles. Internet marketing is increasingly widespread, but researchers emphasize that users do not have access to all relevant medical information [31]. A recent study on supplements intended for cognitive improvement and the prevention of cognitive degeneration [16] showed that 90.9% of the EU products, a substantially higher proportion than in the current study, had at least one unapproved claim. A study analyzing claims of cardioprotective food supplements available for online sale [31] showed the presence of health claims on 40.4% of the analyzed sites, of which 47.8% were authorized claims. In the current study, the proportion of products bearing health claims was more than two-fold higher (86.2%), but the proportion of compliant claims was almost five-fold lower (10.7%). A review of the top 50 websites obtained using Google search for 13 selected common herbal products [32] revealed 13.8% of the websites claiming to diagnose, treat, prevent, or cure a disease, while only 10.5% recommended consulting a healthcare professional before usage, which is similar to the current study, where 9.3% of the products were attributed medicinal properties and 16.1% stated the necessity to consult a doctor or a pharmacist before use. Regardless of the different means of communication discussed in the presented studies as well as purposes and target groups of the analyzed supplements, the findings consistently indicate the need for the stricter control of food (supplement) information to consumers, whether printed on the labels of the products sold “offline” or available “online”. Although there is no clear link between the use of claims and consumer purchasing choices or consumption decisions [36], claims are used by manufacturers to highlight the (unique) benefits of their products [2].

The position of the decision-maker(s) has recently been pointed out by the resolution of the European Parliament (EP) on the implementation of Regulation 1924/2006 on nutrition and health claims made on foods [20], emphasizing legal concerns about the use of the “on-hold” claims. It warns that such practices could mislead consumers, as they may falsely assume that the “on-hold” claims have been scientifically assessed, and highlights the need for action to safeguard consumers. Moreover, as “on-hold” health claims comprise not only those not yet reviewed but also those already assessed negatively, the report calls to reject the latter. It further stresses legislative disparities, market fragmentation, and commercialization of potentially unsafe products due to the absence of an (EU) positive or negative list of botanicals for use in foods (particularly supplements) and of a comprehensive list of their beneficial or harmful health effects. The report [20] also points out the lack of harmonization at the EU level concerning the classification of botanical substances as either food or medicine. The purpose of a medicinal product is to prevent or treat human diseases, while supplements are intended for consumers without immediate medical needs. Therefore, the imperative is to maintain a clear distinction between food and medicine. An inadequate capacity of consumers to distinguish between botanical supplements and traditional herbal medicines based on the same plant substance(s) further complicates the issue. Therefore, the report [20] urges an effective collaboration on establishing a coordinated approach on botanical supplements and an EU-level negative list of botanicals used in food based on their previously identified toxicity and adverse health effects.

The literature also highlights that consumers do not always completely understand the claims on the labels and that the degree of understanding of the claims depends on different consumer characteristics [2,37,38]. The EP report [20] emphasizes that consumers tend to overconsume foods with claims promoting better health; advocates for the inclusion of minimum and maximum consumption thresholds on the foods presenting health claims, in the case of supplements along with a recommendation to consult a healthcare professional, to avoid potential harmful interactions with specific medical treatments; stresses the persistent nature of information asymmetry in a fast-changing food environment; and finally calls for the research into consumer understanding of health claims.

The lack of truthful/transparent warning/precautionary statements can create a misleading impression of safety and universality of usage of a food supplement [16]. Such statements hold significance concerning consumers’ assessment of the suitability of a specific supplement for their individual needs and/or underlying medical conditions, diseases, or allergies to minimize the risk of adverse effects or interactions with other supplements, foods, or medicines [16]. This is particularly important for vulnerable populations such as children and pregnant or lactating women, who are more susceptible to adverse effects. Considering that the majority of the supplements evaluated in the current study were designated for the pediatric population, it is important to recognize and address the heightened concern regarding the well-being of children.

Finally, apart from the evaluation of voluntary claims and (mandatory) statements, several important questions/issues need to be addressed regarding the composition of food supplements, both in terms of labelled ingredients (active and auxiliary substances) and the possible presence of adulterants and/or contaminants. The first point refers to whether the product indeed contains parts/extracts/preparations of labelled herb(s), whether the stated herbal ingredient(s) truly has the potential to exert the claimed effect(s), and whether their doses are adequate to cause the claimed effect(s). The fact that herbal preparation composition could be highly variable, depending on the numerous factors such as plant genetics, growing conditions, climate, soil quality, as well as processing, extraction methods, and/or manufacturing and storage conditions [39], highlights the importance of its standardization. The second concern is that numerous botanicals are reported to contain naturally occurring substances of possible concern for human health when present in food. The mere presence of such a substance in a botanical does not necessarily mean that it will also be present in botanical preparation and, if so, at a dosage needed to cause health concern. Indeed, the plant part used, the method of preparation, and the conditions of use would be the key determining factors of the possible plant-to-preparation transfer. To help with the safety assessment by facilitating hazard identification, EFSA prepared a database known as the Compendium of Botanicals, which has no legal or regulatory force [40], but it should be considered as a part of the EFSA guidance for the safety assessment of botanicals in food [41]. This guidance depends on the available knowledge on a given botanical and the substance(s) it contains, while its extension, the Qualified Presumption of Safety (QPS) approach [39] provides an assessment scheme whose reiterative applications to related botanicals or different botanical preparations obtained from the same plant variety can allow a QPS status to be derived for specific groupings. However, the possibility of establishing QPS status at high taxonomic levels is quite limited due to the particularity of botanicals reflected through their presentation in a wide variety of forms and the high potential of environmental factors to markedly affect their morphology and chemical composition. The next concern is the possibility of product adulteration with unauthorized pharmacologically active compound(s) that provide the claimed effect(s), as it was shown by a rather alarming number of notifications revealed by the recent overview of the RASFF notifications over 2011–2022 [42]. As far as chemical contaminants are concerned, it is important to note that all food supplements evaluated within the scope of the current study went through comprehensive chemical analyses in pursuit of potentially harmful components such as ethanol and residual solvents (solvents are commonly used to prepare herbal extracts) [43], benzene (formed in situ from preserving agent sodium benzoate, which is a labelled ingredient) [44], and an array of toxic metal(loid)s (originating from soil/plant contamination or the supplement production process) [45]. The assessment of health risks associated with the mentioned hazards revealed several issues, highlighting the immense importance of proper quality control in the production process, from the selection of raw materials to the market release of finished products. Product labelling, as a final step, should be a means to facilitate consumers’ choices and serve public health interests.

There is an imperative need to address public health concerns regarding food supplement labelling/advertising, directly communicating health benefits offered to consumers. Addressing these concerns requires engagement and collaboration of all links in the food supplements’ chain, including regulatory authorities and industry and public health professionals on one side and consumers on the other, and all these parties should be held accountable [20]. Ensuring comprehensive safety and quality control, truthful/transparent labelling practices, and a functional nutrivigilance system for reporting adverse reactions of food supplements are essential responsibilities of the former; however, the latter are also expected to make right/informed decisions and choices, albeit on a personal level. To do so, consumers need to be empowered through educational campaigns thoughtfully created to guide them through the future food (supplement) environment [20], which will hopefully be more supportive of consumers’ beliefs and appreciation.

Finally, the current study limitations need to be disclosed and discussed. The focus on specific product forms (liquids, syrups, sprays, drops) limits the multitude of supplements and associated health claims to a feasible number; yet, concomitantly, it restricts the findings’ applicability to the broader spectrum of herbal supplements available in different pharmaceutical forms (e.g., tablets, capsules). The sampling is limited to physical retail outlets (pharmacies, drugstores, health food stores) and excludes online channels. Thus, a wider insight into the real market situation requires the inclusion of other categories of supplements and purchasing channels. All claims, printed on the physical product labels and provided by the advertising media, need to be evaluated. Moreover, information available online, on the manufacturer’s websites, various platforms for online shopping, and social networks needs to be considered.

The listed limitations simultaneously outline the future research directions. However, an investigation into the paradox of poor compliance with regulations despite mandatory pre-market registration in Serbia, i.e., why this system seems ineffective, is beyond the scope of the study. Nevertheless, several possibilities need to be investigated: Is it due to lenient review standards, lack of enforcement post-registration, resource limitations, or other factors? It is up to competent authorities (in Serbian settings, the Ministry of Health) to explore the reasons and ensure an adequate solution to the problem.

In all this, consumers must not be forgotten either, as health claims are meant for them. Indeed, the question of how consumers understand and interpret claims on food supplement labels and how claims/statements influence their personal choices needs to be answered. In short, as for the former, the level of subjectivity decreases with the degree of knowledge on the matter, while the latter is associated with personality characteristics. Consumer science should have its say.

## 5. Conclusions

The study highlights the worrying lack of adherence to food supplement labelling regulations regarding voluntary and mandatory information, despite pre-market registration within a harmonized EU framework (the Serbian context). Such findings confirm that not only do producers circumvent/overstep regulatory requirements, but also that, to be effective, the system of mandatory pre-market registration has to be quality assured. Considering the importance of labelling for consumer protection and public health, the study findings advocate for tightening control of the application of regulatory requirements, foremost in the domain of voluntary information, and an effective solution for on-hold claims on botanicals. It is crucial to explicitly disclose the provisional nature of on-hold health claims and their pending scientific assessment, i.e., to differentiate their regulatory standing from fully authorized claims. By highlighting critical issues, the study has direct implications for consumer protection and public policy, and improving market transparency and regulation; its findings can inform regulatory decision-making and help to create awareness campaigns.

## Figures and Tables

**Table 1:** An overview of provisions of Directive 2002/46/EC [11] and Regulations 1169/2011 [17] and 1924/2006 [18] regarding health claims and precautionary and warning statements on food supplement labels.

N°	Regulation	Provision Type	Provision (Description)
1.	Directive 2002/46/EC [11]	Requirements for label warnings and notes	The food supplements label shall contain a warning not to exceed the stated recommended daily dose, as well as a statement to the effect that food supplements should not be used as a substitute for a varied diet.
		Prohibition	The labelling, presentation, and advertising must not attribute to food supplements the property of preventing, treating, or curing a human disease, or refer to such properties.
2.	Regulation 1169/2011 [17]	Fair information practices	Food information shall not be misleading; food information shall be accurate, clear, and easy to understand for the consumer.
		Prohibition	Food information shall not attribute to any food the property of preventing, treating or curing a human disease, nor refer to such properties.
		Voluntary food information requirements	Food information provided voluntarily shall not mislead the consumer; shall not be ambiguous or confusing for the consumer; shall be based on the relevant scientific data.
3.	Regulation 1924/2006 [18]	Definition of the claim	“Claim means any message or representation, …, including pictorial, graphic or symbolic representation, in any form, which states, suggests or implies that a food has particular characteristics”.
		Definition of the health claim	“Health claim means any claim that states, suggests or implies that a relationship exists between a food category, a food or one of its constituents and health”.
		Definition of the reduction in disease risk claim	“Reduction of disease risk claim means any health claim that states, suggests or implies that the consumption of a food category, a food or one of its constituents significantly reduces a risk factor in the development of a human disease”.
		The use of nutrition and health claims	The use of nutrition and health claims shall not be false, ambiguous or misleading; give rise to doubt about the safety and/or the nutritional adequacy of other foods; encourage or condone excess consumption of a food; state, suggest or imply that a balanced and varied diet cannot provide appropriate quantities of nutrients in general.
		Statement that should follow a health claim	Health claims shall be followed by a statement indicating the importance of a varied and balanced diet and a healthy lifestyle; the quantity of the food and pattern of consumption required to obtain the claimed beneficial effect; a statement addressed to persons who should avoid using the food; a warning for products that are likely to present a health risk if consumed in excess.
4.	EU Register of Health Claims [19]	Inclusion in Register	The use of health claims shall only be permitted if they are authorized and included in the Register of authorized (permitted) health claims.

**Table 2:** Examples of claims presented on the labels of herbal food supplements according to their regulatory compliance.

N	Composition	Compliance	Labelled Claims	“On-hold” Health Claims/*Authorized Claims*
1	β-Glucan elixir (natural bioactive polysaccharide of plant origin), zinc, vitamin C	fully *compliant* (health claims from the Register of authorized claims)	Contains a natural bioactive polysaccharide of plant origin, vitamin C, and zinc.	(Other (non-health) claims)
			*VITAMIN C contributes to the normal function of the immune system, protection of cells from oxidative stress, reduction of tiredness and fatigue.*	*Vitamin C contributes to the normal function of the immune system. Vitamin C contributes to the protection of cells from oxidative stress. Vitamin C contributes to the reduction of tiredness and fatigue.*
			*ZINC contributes to the normal function of the immune system and plays a role in the process of cell division.*	*Zinc contributes to the normal function of the immune system. Zinc plays a role in the process of cell division.*
2	Marshmallow, vitamin C	“on-hold“ claims *compliant* with EFSA Register of questions	For maintaining the health of the respiratory organs.	3723—*Althaea officinalis* L. (Common name: Marshmallow)—Respiratory health: Soothing for mouth and throat/Relieves in case of tickle in the throat and pharynx/Soothing and pleasant effect on throat, pharynx, and vocal cords.
			Marshmallow has a soothing and pleasant effect on the throat, pharynx, and vocal cords.	
			It provides relief in case of throat, pharynx, and vocal cord irritation.	
3	Thyme, primrose, vitamin C	“on-hold“ claims *compliant * with EFSA Register of questions + health claims from the Register of authorized claims	Based on thyme, primrose, and vitamin C.	4258—*Primula veris* (Common Name: Cowslip)—Health of the upper respiratory tract: Promotes upper respiratory tract health.
			Primrose contributes to the relaxation and health of the upper respiratory tract and respiratory health.	4259—*Primula veris* L. syn. Primula officinalis L. (Common name: Cowslip)—Respiratory health: Soothing for mouth and throat/Relieves in case of tickle in the throat and pharynx/Soothing and pleasant effect on throat, pharynx, and vocal cords
				4468—*Primula officinalis* (primrose)-radix—it sustains the respiratory apparatus; saponins have a secretolytic and secretomotor action: it favours expectoration of bronchial secretions.
				3794—*Primula veris* L. em. heids—Contributes to relaxation and mental well-being: Helps to obtain a relaxation effect and regain a natural good temper. Contributes to the recovery of physical and mental well-being.
			Thyme contributes to respiratory health and maintains the normal function of the upper respiratory tract.	2149—*Thymus vulgaris*/*zygis* (Common Name: Thyme)—Health of the upper respiratory tract: Soothing for throat and chest/contributes to wellbeing of chest and throat/contributes to a fresh breath ‘-Good for respiratory tract and/or throat, -Soothes respiratory tract
				2687—Common Thyme (*Thymus vulgaris*, *Thymus zygis*)—Supports secretion of mucus in the upper respiratory tract: Eases expectoration. Helps with a dry cough.
				4167—*Thymus vulgaris* L. (Common name: Thyme)—Respiratory health: Soothing for mouth and throat/Relieves in case of irritation of throat and pharynx/Soothing and pleasant effect on throat, pharynx, and vocal cords
			*Vitamin C contributes to the normal function of the immune system, reducing fatigue and tiredness, and protecting cells from oxidative stress.*	*Vitamin C contributes to the normal function of the immune system. Vitamin C contributes to the reduction of tiredness and fatigue. Vitamin C contributes to the protection of cells from oxidative stress.*
4	Rosehip, primrose	partially NON-COMPLIANT (Register of authorized claims/“on-hold“ claims EFSA Register of questions)	TRADITIONALLY USED AS AN EXPECTORANT AND SUPPORT IN THE TREATMENT OF PRODUCTIVE COUGH.	4258—*Primula veris* (common Name: Cowslip)—Health of the upper respiratory tract: Promotes upper respiratory tract health.
			The active ingredients of primrose (saponins) locally irritate the mucous membrane of the respiratory organs, thereby increasing the secretion of bronchial mucus.	4259—*Primula veris* L. syn*. Primula officinalis* L. (common name: Cowslip)—Respiratory health: Soothing for mouth and throat/Relieves in case of tickle in the throat and pharynx/Soothing and pleasant effect on throat, pharynx and vocal cords
			Saponins reduce the surface tension of mucus, leading to a decrease in the density and viscosity of secretions, thus facilitating expectoration.	4468—*Primula officinalis* (primrose)-radix- it sustains the respiratory apparatus; the saponins have a secretolytic and secretomotor action: it favours expectoration of bronchial secretions.
			PRIMROSE, THROUGH β2 RECEPTORS LOCATED IN THE WALL OF THE RESPIRATORY ORGANS, DILATES THE BRONCHI AND FACILITATES BREATHING;	3680—*Rosa canina* (common Name: Rose Hip)—Respiratory health: helps to soothe common cold/contributes to physical well-being/contributes to the body’s defence
			EXHIBITS ANTIMICROBIAL AND ANTI-INFLAMMATORY EFFECTS.	
5	Lady’s mantle	fully NON-COMPLIANT (Register of authorized health claims/“on-hold“ claims EFSA Register of questions)	BENEFICIALLY ACTS AGAINST INFLAMMATION AND BACTERIA, IN POLYCYSTIC OVARIES, ON REGULATION OF THE SECRETION OF FEMALE HORMONES, BRINGING MENSTRUAL CYCLES IN ORDER.	2203—*Alchemilla vulgaris*—Menstruation: Helps to maintain good comfort before and during the menstrual cycle
			AGAINST DIARRHEA, RESPIRATORY TRACT INFLAMMATION, AND ANEMIA.	2204—*Alchemilla xanthochlora*—common name: Lady’s Manthe—Vascular and Vein Health:/”Used for the good circulation of blood in microvessels”/”Helps to decrease the sensations of heavy legs”.
			TO REDUCE RHEUMATIC PAIN, BLOOD SUGAR LEVEL, AND ELIMINATION OF WATER FROM THE BODY.	2714—*Alchemilla xanthochlora*—common name: Lady’s Manthe—Vascular and Vein Health: “Traditionally used for the good circulation of blood in microvessels”/”Traditionally used to decrease the sensations of heavy legs”/”Used for the good circulation of blood in microvessels”/”Helps to decrease the sensations of heavy legs”.
6	Propolis	fully NON-COMPLIANT (claims for ingredients with no defined “on-hold” claims for specified purposes)	Propolis contains a wide spectrum of PHYSIOLOGICALLY ACTIVE SUBSTANCES such as bioflavonoids, phytohormones, essential oils, pollen, vitamins, micro- and macro-elements.	(No defined “on-hold” claims)
			BACTERIOSTATIC, BACTERICIDAL, ANTIVIRAL, ANTIFUNGAL, IMMUNOSTIMULATORY, AND ANTITUMOR PROPERTIES.	

Green *Italic*—compliant health claims from EU Register of authorized claims; red UPPERCASE—claims noncompliant with “on-hold” claims from EFSA Register of questions.

**Table 3:** The main findings of the studies investigating regulatory adherence of the information related to food supplements communicated through printed product labels/magazines, radio, and TV advertising/internet marketing.

Country	Type of Product (Composition or Intended Use)	Products Bearing Health Claims (N/N, %)	Description	Adherence (N/N, %)	Study/Data Year	Ref.
**Printed labels**						
**Serbia**	multivitamin and mineral	22/48 (46%)	health claims in compliance with the list of approved claims	7/22 (32%)	2011/2010	[23]
			mandatory warning statements (claim about the importance of a varied and balanced diet and a healthy lifestyle)	21/22 (95%)		
			absence of expressions such as “prevention”, “treatment”, or “therapy”, attributing medicinal properties to products	75% of claims		
**Serbia**	omega-3 fatty acid	87/97 (89.7%) any health claim 68/97 (70.1%) claims referring to omega-3 fatty acids	health claims in compliance with the list of approved claims	59/68 (86.8%)	2025/2022–2023	[24]
			mandatory warning statements (claim about the importance of a varied and balanced diet and a healthy lifestyle)	87/97 (89.7%)		
			mandatory warning statement (keep out of reach of children)	76/97 (78.4%)		
			advice not to exceed the recommended daily dose	78/97 (80.4%)		
			warning that the supplement should not be used in children (specified for certain age groups)	34/97 (35.1%)		
			warning about the presence of allergens or sensitivity to certain ingredients/avoid use if an individual is allergic/sensitive to certain ingredients	44/97 (45.4%)		
			A warning requiring consultation with a doctor or pharmacist	48/97 (49.5%)		
**USA**	Ayurvedic herbal	structure/function and general well-being claims: 35/51 (68.6%) physical product labels; 32/42 (76.2%) online listing	at least one U.S. regulatory noncompliance	31/51 (60.8%)	2023/2021	[26]
			missing/noncompliant disclaimers for structure/function or general well-being claims	17/51 (33.3%)		
			missing/noncompliant “Supplement Facts” label	15/51 (29.4%)		
			noncompliant statement of identity	14/51 (27.5%)		
			missing/noncompliant domestic mailing address or phone number	13/51 (25.5%)		
			disease claims	4/51 (7.8%) of physical labels; 16/42 (38.1%) of online listings		
**Slovenia**	resveratrol	8/20 (40%)	health claims compliant with the list of approved claims	8/8 (100%)	2023/2022	[25]
			the mandatory warning statements (claim emphasizing the importance of a varied and balanced diet and a healthy lifestyle)	20/20 (100%)		
			the warning for products that are likely to present a health risk if consumed in excess	20/20 (100%)		
			the warning for persons who should avoid the food	13/20 (65%)		
**USA**	weight loss and muscle building	6.5 (SD 2.5) claims per product	absence of a promising weight loss statement despite the lack of evidence to support such claims	67/110 (60.9%) reduce body fat, BMI, promote weight loss; 35/110 (31.8%) guarantees success; 33/110 (30.0%) suppresses hunger, decreases appetite; 28/110 (25.5%) quick weight loss; 25/110 (22.7%) boosts metabolism	2021/2013	[27]
			the presence of the FDA disclaimer	59/110 (53.6%)		
			the presence of warnings for vulnerable populations (children, pregnant or nursing women…)	62/110 (56.4%)		
			the presence of information on side effects	67/110 (60.9%)		
			absence of performance claims (fast acting, long-lasting…)	30/110 (27.3%)		
**Magazine, radio, and TV advertising**						
**Poland** **(radio, TV)**	dietary supplements	46 products (76 health claims)	compliance with health/legal considerations within the framework of the EU regulation and the Polish regulation between TV broadcasting companies and supplement manufacturers	46/46 (100%)	2022/2020	[28]
			absence of unsubstantiated claims regarding effectiveness (promising weight loss or memory improvement)	33/46 (71.8%)		
**Spain** **(radio)**	food supplements	437 advertisements; 100% function claims; 89/437 (20.4%) prohibited disease claims; 38/437 (8.7%) reduction in disease risk claims	the presence of claims and supplement ingredients authorized by EFSA	19.7% function claims; 79.6% disease claims; 0% reduction in disease risk claims	2021/2017	[29]
**USA** **(magazines)**	dietary supplements	5350 structure–function claims/6179 advertisements (86.6%); 2.5 claims per ad	absence of verbs indicating a disease treatment/cure effect	(a significant number)	2017/2003–2009	[30]
			absence of unsubstantiated validity claims that a product is “scientifically proven” or “guaranteed”	79.5%		
**Internet marketing**						
**Google, Yahoo, Bing (Serbia)**	cognitive improvement/degeneration prevention	75/75 (100%)	the presence of the FDA disclaimer	45/49 (91.8%) USA products	2024/2023	[16]
			absence of unapproved claims	1/11 (9.1%) EU products		
			the presence of obligatory warnings	31/75 (58.7%)		
			the period of use specified	8/75 (10.7%)		
			the presence of precautions for overdose	24/75 (32.0%)		
			the precautions for vulnerable population(s)	45/75 (60.0%)		
			the presence of information on potential adverse effects	58/75 (77.3%)		
**Google, Yahoo, Bing** **(Serbia)**	cardioprotective supplements (omega-3 fatty acids based)	23/57 (40.4%) risk reduction claims 50/57 (87.7%) structure/function claims	the presence of claims authorized according to the FDA	11/23 with claims (47.8%)	2020/2018	[31]
			the presence of the FDA disclaimer	34/57 (68.0%)		
			the presence of information on the adverse effects	1/57 (1.8%)		
			the presence of information on possible interactions	12/57 (21.0%)		
			warnings regarding the use of the product	38/57 (66.7%)		
**Google** **(USA)**	common herbal products	13 products (top 50 websites of each product)	absence of claims related to the properties of treatment, prevention, or cure of a disease	560/650 (86.2%)	2014/2012–2013	[32]
			the presence of the FDA disclaimer	54/650 (8.3%)		
			recommendation to consult a healthcare professional before usage	68/650 (10.5%)		
			the presence of the information on safety (adverse effects, interaction, etc.)	<8%		
			the presence of specific safety information related to the use by pregnant or lactating individuals	46/650 (7.0%)		
**Seznam, Google, Centrum** **(Czech R.)**	top-selling food supplements	100 supplements (199 web domains and 850 websites)	absence of claims related to the properties of treatment, prevention, or cure of a disease	778/850 (91.5%)	2018/2014	[33]
			absence of unauthorized health claims	395/850 (46.5%)		

## Data Availability

The original contributions presented in this study are included in the article/Supplementary Materials. Further inquiries can be directed to the corresponding author.

## Supplementary Materials

The following supporting information can be downloaded at: https://www.mdpi.com/article/10.3390/nu17091571/s1, Table S1: Herbal food supplement sample information; Table S2: Labels of the herbal food supplements without labelled health claims; Table S3: Labels of the herbal food supplements which carried only the health claims from the list of authorized health claims; Table S4: Labels of herbal food supplements which carried “on-hold” claims in compliance with “on-hold” claims from EFSA Register of questions; Table S5: Labels of herbal food supplements which carried claims partially noncompliant with List of authorized health claims and “on-hold” claims from EFSA Register of questions; Table S6: Labels of herbal food supplements which carried claims fully noncompliant with List of authorized health claims and “on-hold” claims from EFSA Register of questions; Table S7: Labels of herbal food supplements which carried health claims for ingredients for which there were no defined “on-hold” claims from EFSA Register of questions for specified purposes; Table S8: Precautionary and warning statements on the supplement’s usage listed on the labels of herbal food supplements.
