# Supplementary material for: Landscape of Herbal Food Supplements: Where Do We Stand with Health Claims?
Source: Nutrients. 2025 May 2;17(9):1571. doi: 10.3390/nu17091571 (PMC12073160; doi:10.3390/nu17091571)
Supplement: Supplementary file 1 [file nutrients-17-01571-s001.zip › nutrients-3606592-supplementary.pdf]

Supplementary material

Landscape of herbal food supplements labelling: Where do we stand with health claims?

Slađana Vojvodić [1], Dunja Kobiljski [2], Branislava Srđenović Čonić [1,3] and Ljilja Torović [1,3,*]

[1] Department of Pharmacy, Faculty of Medicine, University of Novi Sad, Hajduk Veljkova 3, 21000 Novi Sad, Serbia; sladja.vojvodic@uns.ac.rs (S.V.); branislava.srdjenovic-conic@mf.uns.ac.rs (B.S.Č.)

[2] Department of Industrial Engineering and Management, Faculty of Technical Sciences, University of Novi Sad, Trg Dositeja Obradovića 6, 21000 Novi Sad, Serbia; dunjakobiljski@uns.ac.rs

[3] Center for Medical and Pharmaceutical Investigations and Quality Control, Faculty of Medicine, University of Novi Sad, Hajduk Veljkova 3, 21000 Novi Sad, Serbia

* Correspondence: ljilja.torovic@mf.uns.ac.rs

Table legend		Page
**Table S1**	Herbal food supplement sample information	2
**Legend**	The legend for Tables S2-S7	6
**Table S2**	Labels of the herbal food supplements without labelled health claims	7
**Table S3**	Labels of herbal food supplements which carried only the health claims from the List of authorized health claims	8
**Table S4**	Labels of herbal food supplements which carried 'on-hold' claims in compliance with 'on hold' claims from the EFSA Register of questions	10
**Table S5**	Labels of herbal food supplements which carried claims partially non-compliant with the List of authorized health claims and 'on-hold' claims from the EFSA Register of questions	23
**Table S6**	Labels of herbal food supplements which carried claims fully non-compliant with the List of authorized health claims and 'on-hold' claims from the EFSA Register of questions	37
**Table S7**	Labels of herbal food supplements which carried health claims for ingredients for which there were no defined 'on-hold' claims from the EFSA Register of questions for the specified purposes	43
**Table S8**	Precautionary and warning statements on the supplement's usage listed on the labels of herbal food supplements	44

Table S1. Herbal food supplement sample information.

Code	Sample Name	Manufacturer	Ingredients	Package (ml)	Population group (years)
**1**	Pulmint Timal	ESENSA, Serbia	thyme, primrose, rose hip, basil	200	adults children >12
**2**	Pulmint Primal	ESENSA, Serbia	primrose, marshmallow, chamomile	200	adults children >12
**3**	Protect IMUNOβETA	ESENSA, Serbia	β-glucan, zinc, vitamin C	171.3	children >3
**4**	EXPULMO DASA with plantago	ESENSA, Serbia	plantago	111.5	children >1
**5**	EXPULMO ZMAJAC with primrose and thyme	ESENSA, Serbia	primrose, thyme, rose hip	111.3	children >1
**6**	HERBIKO Natural Primrose	Abela Pharm, Serbia	rose hip, primrose	125	adults children >4
**7**	HERBIKO Natural Plantago	Abela Pharm, Serbia	rose hip, plantago	125	adults children >3
**8**	HERBIKO with honey for children	Abela Pharm, Serbia	marshmallow, chamomile, rose hip, basil	125	children >1
**9**	Syrup MARSHMALLOW with chamomile and vitamin C	Sinefarm, Serbia	marshmallow, chamomile, vitamin C	113.6	adults children >4
**10**	Syrup PRIMROSE with thyme and vitamin C	Sinefarm, Serbia	primrose, thyme, vitamin C	113.6	adults children >4
**11**	Mucoplant syrup for good night, oral solution	Dr. Theiss, Germany	plantago, thyme, chamomile, lemon balm, vitamin C	100	adults children >4
**12**	BRNOHI HERB	KIRKOLINA Plus, Serbia	marshmallow, chamomile, honey	200	adults children >4
**13**	EXPERTO HERB	KIRKOLINA Plus, Serbia	primrose, thyme, honey	200	adults children >4
**14**	IMMUNO	Pharmalife Research, Italy	rose hip, astragalus, Siberian ginseng, vitamin E, vitamin B6, zinc, copper	200	children >1
**15**	BRONCAMIL	Pharmalife Research, Italy	sunflower, immortelle, plantago, scots pine, eucalyptus, thyme	200	children >1 adults
**16**	OMEGA JUNIOR	Pharmalife Research, Italy	blackcurrant, soy, vitamin E, vitamin B6	30	children from birth
**17**	IMMUNIFLOR	ESI, Italy	blueberry, acerola, rose hip, zinc, probiotics	200	children >4
**18**	BIMUNAL IMUNO for you	4U pharma, Switzerland	baobab, elder, acerola, vitamin C, zinc, copper, vitamin D	300	children >1
**19**	VIRANTO for you	4U pharma, Switzerland	astragalus, elder, acerola, zinc, copper, vitamin D	100	children >1
**20**	ARONIA herbal drops	DEVERRA, Serbia	aronia	30	adults
**21**	BRONHIKAP herbal drops	DEVERRA, Serbia	plantago, chamomile, basil, coltsfoot	30	adults
**22**	FOR APPEASEMENT herbal drops	DEVERRA, Serbia	lemon balm, hops, St John's-wort, valerian, rosemary	30	adults
**23**	DEPRESINE herbal	DEVERRA, Serbia	St John's-wort, mint, lemon	30	adults

	drops		balm, Breckland thyme, basil, rosemary, pot marigold		
**24**	RELAXKAP herbal drops	DEVERRA, Serbia	valerian, lemon balm, hawthorn, lavender, St John's-wort, basil	30	adults
**25**	LADY'S MANTLE herbal drops	DEVERRA, Serbia	Lady's mantle	30	adults
**26**	UVA herbal drops	DEVERRA, Serbia	uva-ursi	30	adults
**27**	Herbal drops LADY'S MANTLE	Vitalia Farm, Serbia	Lady's mantle	30	adults
**28**	PROPOLIS EXTRA DROPS	NATURALIS, Serbia	propolis	20	adults
**29**	Propomel drops for children	ESENSA, Serbia	propolis	30	children >4
**30**	Marshmallow	TGFarm Medico, Serbia	marshmallow, thyme, menthol, vitamin C	125	adults<br>children >4
**31**	BABY marshmallow	Hemofarm, Serbia	marshmallow, vitamin C	125	children >3
**32**	Altiprim P	Institute "Dr Josif Pančić", Serbia	marshmallow, thyme, primrose, honey	100	children >1
**33**	MAXOGAST	MaxMedica, Serbia	chamomile, wild candytuft, greater celandine, liquorice, caraway, lemon balm, angelica, milk thistle, mint	50	adults
**34**	Bronhoklir	Hemofarm, Serbia	marshmallow	200	adults<br>children >3
**35**	HERBIKO PropoMucil	Abela Pharm, Serbia	marshmallow, honey, propolis, primrose, mint	120	adults<br>children >7
**36**	Salvit Beta glucan	SALVUS, Poland	beta-glucan, zinc, elder	150	children >1<br>adults
**37**	ŽAK	Hemofarm, Serbia	thyme, primrose, vitamin C	125	children >4
**38**	GAS	Pharmalife Research, Italy	fennel, chamomile, caraway, mint	30	children from birth
**39**	FERRO C	Pharmalife Research, Italy	iron, zinc, copper, vitamin C, vitamin B12, chamomile, acerola	200	children >1
**40**	ISILAX	Pharmalife Research, Italy	manna ash, plum, apple, cheeses, inulin, apple pectin	200	children >1<br>adults
**41**	APPETITO	Pharmalife Research, Italy	wheat germ, pollen, great yellow gentian, common centaury, fenugreek	200	children >1<br>adults
**42**	Vendoksin drops	ALTERNATIVA MEDICA (ALTERA MEDICA), Serbia	chestnut, yarrow, stork's bill, yellow sweet clover, white sweet clover, pot marigold	50	adults
**43**	Floravitex drops	ALTERNATIVA MEDICA (Mediflora), Serbia	vitex	50	adults
**44**	FemiSan A	HERBASVET, Serbia	Lady's mantle, yarrow, pot marigold, bigroot geranium, raspberry, parsley	30	adults
**45**	FemiSan B	HERBASVET, Serbia	mistletoe, lemon balm, yarrow, pot marigold, valerian	30	adults
**46**	Echinaid	ESI, Italy	echinacea	50	adults<br>children >12
**47**	EXPULMO LUNAC sa with marshmallow	ESENSA, Serbia	marshmallow, chamomile, rose hip	112.1	children >1
**48**	EXPULMO PROSPERA with ivy	ESENSA, Serbia	common ivy	114.9	children >4
**49**	VIRANTO Forte for	4U pharma,	bigroot geranium, elder, acerola,	100	children >3

	you	Switzerland	dog rose, zinc, vitamin D, copper		adults
**50**	Mucoplant plantago syrup with echinacea and vitamin C	Dr. Theiss, Germany	plantago, echinacea, vitamin C	100	adults children >4
**51**	Bronhoklir for smokers	Hemofarm, Serbia	thyme, primrose, echinacea, vitamins C,E,A	200	adults
**52**	Syrup PLANTAGO C	Sinefarm, Serbia	plantago, thyme, primrose, vitamin C	116.8	adults
**53**	TUSSIFLUX BIMBI	Pharmalife Research, Italy	Iceland lichen, plantago, propolis, cheeses, scots pine, elder	200	children >4
**54**	Pulmint tusik	ESENSA, Serbia	N-acetylcysteine, propolis, thyme	200	adults
**55**	Floradix	SALUS Haus, Germany	iron, vitamins B1,B2,B6,B12,C, carrot, nettle, spinach, common couch, fennel, brown algae, hibiscus	250	children >1 adults
**56**	Floradix - Kindervital for children	SALUS Haus, Germany	calcium, vitamins A,B,C,D,E, carrot, coriander, watercress, mint, spinach, chamomile	250	children >4
**57**	Alpikol Imuno syrup	Alpen Pharma, Poland	elder, beta glucan, bigroot geranium	120	children >7 adults
**58**	PROPOLIS DROPS	MAJANA, Serbia	propolis	20	adults children >4
**59**	Propolis drops	ProPolisPharm, Serbia	propolis	20	adults
**60**	Ferrolin C	ESI, Italy	iron, vitamin C from acerola, folic acid	20	adults
**61**	Calma-B	ERBA VITA	linden, lemon balm, chamomile, passion flowers, sweet orange, lavender	150	children >4
**62**	Immuno-B	ERBA VITA, San Marino	echinacea, dog rose, zinc, copper	150	adults
**63**	X-IMMUNO KID	AORA HEALTH (Rioja Nature Pharma), Spain	beta-glucan from yeast, spirulina, vitamins C,D, minerals selenium, zinc, iodine	150	children >4
**64**	Martians PROimun ACUTE	WALMARK, the Czech Republic	elder, rose hip, beta-glucan, rutin, vitamin C	150	children >3
**65**	Imuno glucan	MEDIS, Slovenia	immunoglucan, vitamin C	120	children >1 adults
**66**	NEFRO VIT herbal drops	HERBASVET, Serbia	speedwell, dandelion, woundwort, birch, field horsetail	30	adults
**67**	GREEN FIT herbal drops	WELLGREEN, Serbia	fennel, alder buckthorn, field horstail, birch, ginger	50	adults
**68**	GINKOBILOBA drops	Sinefarm, Serbia	ginkgo biloba	30	adults
**69**	Propolis with plantago and vitamin C	Sinefarm, Serbia	propolis, plantago, vitamin C	20	adults
**70**	Propolis extra 25%	Sinefarm, Serbia	propolis	20	adults
**71**	Propolis Ginseng drops	ProPolisPharm, Serbia	propolis, ginseng	20	adults
**72**	Propolis drops for children	ProPolisPharm, Serbia	propolis	20	children >4
**73**	Propolis C drops	ProPolisPharm, Serbia	propolis, vitamin C	20	adults
**74**	Propolis M drops	ProPolisPharm, Serbia	propolis, royal jelly	20	adults
**75**	Pulmint Altal	ESENSA, Serbia	marshmallow, cheeses, basil	200	adults

					children >12
**76**	HERBIKO Natural Marshmallow	Abela Pharm, Serbia	marshmallow, rose hip	125	adults<br>children >3
**77**	HERBIKO with honey	Abela Pharm, Serbia	marshmallow, primrose, chamomile, basil, vitamin C	125	adults<br>children >3
**78**	HERBIKO PropoMucil for children	Abela Pharm, Serbia	marshmallow, honey, propolis, rose hip	120	children >3
**79**	HERBIKO	Abela Pharm, Serbia	marshmallow, primrose, chamomile, basil, vitamin C	250	adults<br>children >3
**80**	IMMUNOTROFINA	DMG Italia, Italy	arginine, vitamins B5,B6,B12,D3, iodine, beta-glucan	200	adults
**81**	Hawthorn drops	Institute "Dr Josif Pančić", Serbia	hawthorn	30	adults
**82**	Common nettle drops	Institute "Dr Josif Pančić", Serbia	nettle	30	adults
**83**	Wild garlic drops	Institute "Dr Josif Pančić", Serbia	wild garlic	30	adults
**84**	Valerian drops	Institute "Dr Josif Pančić", Serbia	valerian	30	adults
**85**	Garlic drops	Institute "Dr Josif Pančić", Serbia	garlic	30	adults
**86**	Arkoroyal junior	Arkopharma, France	honey, royal jelly	300	children >3
**87**	Arkoroyal synergie	Arkopharma, France	ginseng, royal jelly, acerola, propolis	200	adults<br>children >15

The legend for Tables S2-S7.

Legend	Composition	Purpose/effect on the organ system	Claims from the labels of herbal food supplements	′on hold′ health claims / authorised health claims
	No health claims labeled	not declared		No health claims labeled
		other claims	Other (non-health) claims	Other (non-health) claims
	Claims in compliance with the List of authorised health claims	immune system	Claims in compliance with the List of authorised health claims	Authorised health claims
	′on hold′ claims	respiratory system	Claims/parts of claims in compliance with ′on hold′ claims	**′on hold′** claims (EFSA Register of questions)
	Not in compliance with regulations	antimicrobial effect	Claims/parts of claims not in compliance with regulations	Claims/parts of claims not in compliance with regulations
		anti-inflammatory effect		No "on hold" claims defined
		antiseptic effect		
		antibiotic effect		
		antitumor effect		
		antioxidative effect		
		nervous system		
		gastrointestinal tract (GIT)		
		endocrine system		
		urogenital system		
		musculo-skeletal system		
		cardiovascular system (CVS)		
		multiple systems of organs		

Table S2. Labels of herbal food supplements without labelled health claims.

N°	Composition	Purpose/effect on the organ system	Claims from the labels of herbal food supplements	′on hold′ health claims / authorised health claims
1	thyme, primrose, rose hip, basil			No health claims labelled
2	primrose, marshmallow, chamomile			No health claims labelled
4	plantago			No health claims labelled
5	primrose, thyme, rose hip			No health claims labelled
16	blackcurrant, soy, vitamin E, vitamin B6		Food supplement for infants and young children with blackcurrant oil, soybean oil, and vitamins E and B6.<br><br>Blackcurrant oil and soybean oil are a source of essential polyunsaturated fatty acids: linoleic and alpha-linolenic.	No health claims labelled
27	Lady’s mantle			No health claims labelled
28	propolis		Food supplement based on ethanolic extract of propolis.	No health claims labelled
29	propolis		Dietary product, liquid food supplement based on propolis.	No health claims labelled
47	marshmallow, chamomile, rose hip			No health claims labelled
75	marshmallow, cheeses, basil			No health claims labelled
86	honey, royal jelly			No health claims labelled
87	ginseng, royal jelly, acerola, propolis			No health claims labelled

Table S3. Labels of herbal food supplements which carried only the health claims from the List of authorized health claims.

N°	Composition	Purpose/effect on the organ system	Claims from the labels of herbal food supplements	′on hold′ health claims / authorised health claims
3	β-glucan elixir (natural bioactive polysaccharide of plant origin), zinc, vitamin C		The product contains a natural bioactive polysaccharide of plant origin, vitamin C, and zinc.	Other (non-health) claims
		immune	VITAMIN C contributes to the normal function of the immune system, protection of cells from oxidative stress, reduction of tiredness and fatigue.	Vitamin C contributes to the normal function of the immune system. Vitamin C contributes to the protection of cells from oxidative stress. Vitamin C contributes to the reduction of tiredness and fatigue.
		immune	ZINC contributes to the normal function of the immune system and plays a role in the process of cell division.	Zinc contributes to the normal function of the immune system. Zinc plays a role in the process of cell division.
36	beta-glucan, zinc, elder	immune	Strong and resistant	
			Food supplement for children older than 3 years and adults with beta-glucan, zinc and elder fruit juice.	
		immune	Zinc contributes to the normal function of the immune system.	Zinc contributes to the normal function of the immune system.
38	iron, zinc, copper, vitamin C, vitamin B12, chamomile, acerola		Food supplement for children and adults with herbal extracts, minerals, iron, zinc, copper and vitamins C and B12.	
		CVS	Iron contributes to normal formation of haemoglobin.	Iron contributes to normal formation of red blood cells and haemoglobin.
		CVS	Copper contributes to normal iron transport in the body.	Copper contributes to normal iron transport in the body.
		CVS	Vitamin C increases iron absorption.	Vitamin C increases iron absorption.
60	iron, vitamin C from acerola, folic acid		Food supplement representing an additional source of iron, vitamin C from acerola and folic acid in the form of an oral solution. It is intended for individuals who require increased intake of these substances due to deficiency or increased bodily needs.	
		multiple systems of organs	Iron contributes to normal formation of red blood cells and haemoglobin, as well as normal oxygen transport in the body.	Iron contributes to normal formation of red blood cells and haemoglobin. Iron contributes to normal oxygen transport in the body.
			Vitamin C contributes to normal iron absorption and the protection of cells from oxidative stress.	Vitamin C increases iron absorption. Vitamin C contributes to the protection of cells from oxidative stress.
			Folic acid contributes to maternal tissue growth during pregnancy and contributes to normal blood cells formation.	Folate contributes to maternal tissue growth during pregnancy. Folate contributes to normal blood formation.

			Low maternal folate status is a risk factor in the development of neural tube defects in the developing foetus.	Low maternal folate status is a risk factor in the development of neural tube defects in the developing foetus.
			Vitamin C and folic acid contribute to the reduction of tiredness and fatigue.	Vitamin C contributes to the reduction of tiredness and fatigue. Folate contributes to the reduction of tiredness and fatigue.
33	beta-glucan from yeast, spirulina, vitamins C,D, minerals selenium, zinc, iodine		Food supplement containing beta-glucans from yeast, spirulina algae, vitamins and minerals, intended for children aged 3 years and older.	
		immune	Vitamin D contributes to the normal function of the immune system of children.	Vitamin D contributes to the normal function of the immune system of children.
		growth	Iodine contributes to the normal growth of children.	Iodine contributes to the normal growth of children.
65	immunoglucan, vitamin C	immune	Vitamin C contributes to the normal function of the immune system.	Vitamin C contributes to the normal function of the immune system.
73	propolis, vitamin C	immune	The product is intended to strengthen the body.	
		immune	Vitamin C contributes to the normal function of the immune system.	Vitamin C contributes to the normal function of the immune system.
			The product is not a medicine nor a diagnostic device.	
80	arginine, vitamins B5,B6,B12,D3, iodine, beta-glucan		Food supplement that represents an additional source of beta-glucans, arginine, vitamins B5, B6, B12, vitamin D, and iodine.	
			These ingredients have important physiological roles in the body, such as:	
		immune	Vitamins B6, B12 and D contribute to the normal function of the immune system.	Vitamin B6 contributes to the normal function of the immune system. Vitamin B12 contributes to the normal function of the immune system. Vitamin D contributes to the normal function of the immune system.
		immune	Vitamins B5, B6 and B12 contribute to the reduction of tiredness and fatigue.	Pantothenic acid contributes to the reduction of tiredness and fatigue. Vitamin B6 contributes to the reduction of tiredness and fatigue. Vitamin B12 contributes to the reduction of tiredness and fatigue.
		musculo-skeletal	Vitamin D contributes to normal absorption of calcium and phosphorus, as well as maintenance of normal muscles, bones and teeth.	Vitamin D contributes to normal absorption/utilisation of calcium and phosphorus. Vitamin D contributes to the maintenance of normal muscle function. Vitamin D contributes to the maintenance of normal bones. Vitamin D contributes to the maintenance of normal teeth.
		endocrine	Iodine contributes to the normal production of thyroid hormones and normal thyroid function.	Iodine contributes to the normal production of thyroid hormones and normal thyroid function.
			Arginine is an amino acid involved in protein synthesis. Beta-glucan is a nutrient isolated from yeast Saccharomyces cerevisiae. It is recommended in case of deficiency or increased needs for intake of these ingredients in the body.	

Table S4. Labels of herbal food supplements which carried ′on-hold′ claims in compliance with ′on hold′ claims from the EFSA Register of questions.

N°	Composition	Purpose/effect on the organ system	Claims from the labels of herbal food supplements	′on hold′ health claims / authorised health claims
11	plantago, thyme, chamomile, lemon balm, vitamin C	respiratory	Food supplement with a blend of herbal extracts (plantago, thyme, chamomile and lemon balm) traditionally used to preserve respiratory system function and alleviate cough accompanied by excessive mucus production.	
		immune	Vitamin C contributes to the normal function of the immune system.	Vitamin C contributes to the normal function of the immune system.
				**3791 - Plantago lanceolata L. (Common name: Ribwort plantain) - Respiratory health:** Soothing for mouth and throat / Reliefs in case of tickle in the throat and pharynx / Soothing and pleasant effect on throat, pharynx and vocal cords<br>**3312 - Chamomile (Matricaria chamomilla L.) - Respiratory health:** Release of the respiratory tract - Supportive and soothing in case of dry cough, tickle in the throat - Soothing the throat - Respiratory comfort - Helps to soften respiratory troubles like coughs and sore throat in a natural way - Helps maintain respiratory health<br>**3313 - Chamomilla recutita (L.) (Chamomile-extract) – relief of airways with common cold:** relief of airways caused by common cold.<br>**3443 - Matricaria recutita L. (Common name: Chamomile) - Respiratory health:** Soothing for mouth and throat / Reliefs in case of tickle in the throat and pharynx / Soothing and pleasant effect on throat, pharynx and vocal cords<br>**4079 - Matricaria recutita L. (Common name: Chamomile) - Respiratory health:** Soothing for mouth and throat / Reliefs in case of irritation of throat and pharynx / Soothing and pleasant effect on throat, pharynx and vocal cords<br>**2149 - Thymus vulgaris/zygis (Common Name : Thyme) - Health of the upper respiratory tract:** Soothing for troat and chest /contributes to wellbeing of chest and throat /contributes to a fresh breath '-Good for respiratory tract and/or throat, -Soothens the respiratory tract<br>**2687 - Common Thyme (Thymus vulgaris, Thymus zygis) - Supports secretion of mucus in the upper respiratory tract:** Eases expectoration. Helps with dry cough.<br>**4167 - Thymus vulgaris L. (Common name: Thyme) - Respiratory health:** Soothing for mouth and throat / Reliefs in case of irritation of throat and pharynx / Soothing and pleasant effect on throat, pharynx and vocal cords<br>**2303 - Melissa officinalis L. (Common name: Lemon balm) - Respiratory health:** Soothing for mouth and throat / Reliefs in case of tickle in the throat and pharynx / Soothing and pleasant effect on throat, pharynx and vocal cords

14	rose hip, astragalus, Siberian ginseng, vitamin E, vitamin B6, zinc, copper	immune	Food supplement for children with herbal extracts, vitamins E and B6 and minerals zinc and copper for the normal function of the immune system.	Zinc contributes to the normal function of the immune system.
		immune	Zinc, copper, vitamin B6, astragalus root and Siberian ginseng root contribute to the normal function of the immune system.	Copper contributes to the normal function of the immune system.<br>Vitamin B6 contributes to the normal function of the immune system.
		immune	Vitamin E contributes to the protection of cells from oxidative stress.	Vitamin E contributes to the protection of cells from oxidative stress.
				**3259 - ASTRAGALUS MEMBRANACEUS BUNG. - Contributes to physical well-being:** Helps maintaining mobility and flexibility of joints. Contribute to the resistance during the premenstrual cycle. Contributes to relieve the menopause symptoms.<br>**3735 - Astragalus membranaceus (Common Name : Milk-vetch) - Immune health:** Supports the natural defences<br>**3736 - ASTRAGALUS MEMBRANACEUS BUNG. - Contributes to body defences against external agents:** Increases the physiological resistance of the organism in case of severe ambiance conditions.<br>**3968 - Astragalus membranaceus (Common Name : Milk vetch) - Immune health:** Supports the natural defences<br>**3704 - Eleutherococcus senticosus = Acanthopanax senticosus (Common Name : Taigaroot, Siberian ginseng) - Immune health:** Support of the body´s defence /contributes to the /supports the immune system<br>**3682 - Rosa canina (Common Name : Rose Hip) - Immune health:** Contributes to the resistance against health precarious microorganism/contributes to physical wel-being
30	marshmallow, thyme, menthol, vitamin C	respiratory	For a healthy upper respiratory tract mucos membranes and increased body resistance.	**3723 - Althaea officinalis L. (Common name: Marshmallow) - Respiratory health:** Soothing for mouth and throat / Reliefs in case of tickle in the throat and pharynx / Soothing and pleasant effect on throat, pharynx and vocal cords
		respiratory	The marshmallow root and thyme herb contribute to reducing of irritation of throat and pharynx.	**4070 - Levomentholum (Common name: Menthol) - -Respiratory Health:** Soothing for mouth and throat / Reliefs in case of irritation of throat and pharynx / Soothing and pleasant effect on throat, pharynx and vocal cords
		immune	Vitamin C contributes to the normal function of the immune system.	**2675 - Menthol - Influense of nasal airflow:** Helps to improve nasal airflow.<br>**3422 - Levomentholum - Respiratory Health:** Soothing for mouth and throat / Reliefs in case of tickle in the throat and pharynx / Soothing and pleasant effect on throat, pharynx and vocal cords<br>**2149 - Thymus vulgaris/zygis (Common Name : Thyme) - Health of the upper respiratory tract:** Soothing for troat and chest /contributes to wellbeing of chest and throat /contributes to a fresh breath '-Good for respiratory tract and/or throat, -Soothens the respiratory tract<br>**2687 - Common Thyme (Thymus vulgaris, Thymus zygis) - Supports secretion of mucus in the upper respiratory tract:** Eases expectoration. Helps with dry cough.

				**4167 - Thymus vulgaris L. (Common name: Thyme) - Respiratory health:** Soothing for mouth and throat / Reliefs in case of irritation of throat and pharynx / Soothing and pleasant effect on throat, pharynx and vocal cords<br>**2150 - Thymus vulgaris (Common Name : Thyme) - Immune health**<br>Vitamin C contributes to the normal function of the immune system.
37	thyme, primrose, vitamin C		Food supplement based on thyme, primrose and vitamin C.	**4258 - Primula veris (Common Name: Cowslip) - Health of the upper respiratory tract:** Promotes upper respiratory tract health.
		respiratory	Thyme contributes to respiratory health and maintaining the normal function of the upper respiratory tract.<br><br>Primrose contributes to relaxation and health of the upper respiratory tract and respiratory health.	**4259 - Primula veris L. syn. Primula officinalis L. (Common name: Cowslip) - Respiratory health:** Soothing for mouth and throat / Reliefs in case of tickle in the throat and pharynx / Soothing and pleasant effect on throat, pharynx and vocal cords<br>**4468 - Primula officinalis (promrose)-radix- - it sustain the respiratory apparatus; the saponins have a secretolytic and secretomotor action**: it favours expectoration of bronchial secretions.
		immune	Vitamin C contributes to the normal function of the immune system, reducing fatigue and tiredness, and protecting cells from oxidative stress.	**3794 - PRIMULA VERIS L. EM.HEIDS - Contributes to relaxation and mental well-being:** Helps to obtain a relaxation effect and regain a natural good temper. Contributes to recover physical and mental well-being.<br>**2149 - Thymus vulgaris/zygis (Common Name : Thyme) - Health of the upper respiratory tract:** Soothing for troat and chest /contributes to wellbeing of chest and throat /contributes to a fresh breath '-Good for respiratory tract and/or throat, -Soothens the respiratory tract<br>**2687 - Common Thyme (Thymus vulgaris, Thymus zygis) - Supports secretion of mucus in the upper respiratory tract:** Eases expectoration. Helps with dry cough.<br>**4167 - Thymus vulgaris L. (Common name: Thyme) - Respiratory health:** Soothing for mouth and throat / Reliefs in case of irritation of throat and pharynx / Soothing and pleasant effect on throat, pharynx and vocal cords<br>Vitamin C contributes to the normal function of the immune system. Vitamin C contributes to the reduction of tiredness and fatigue. Vitamin C contributes to the protection of cells from oxidative stress.
50	plantago, echinacea, vitamin C	respiratory	Food supplement for cough and discomfort in the respiratory tract.	**3791 - Plantago lanceolata L. (Common name: Ribwort plantain) - Respiratory health:** Soothing for mouth and throat / Reliefs in case of tickle in the throat and pharynx / Soothing and pleasant effect on throat, pharynx and vocal cords
		respiratory	Food supplement based on a blend of plant extracts that has a beneficial effect on the mucous membranes of the mouth and throat, as well as the respiratory tract.	**4096 - Plantago lanceolata L. (Common name: Ribwort plantain) - Respiratory Health:** Soothing for mouth and throat / Reliefs in case of irritation of throat and pharynx / Soothing and pleasant effect on throat, pharynx and vocal cords
		immune	Enriched with vitamin C which contributes to the normal function of the immune system.	**3950 - Echinacea purpurea (Common name: Purple Coneflower Herb) - Healt of the upper respiratory tract:** adjuvant terapy of recurrent infection of the upper respiratory tract/prophylaxis of recurrent infection of the upper respiratory tract/supportive therapy for cold<br>**4032 - Echinacea purpurea L. Moench (Common name: Echinacea) - Respiratory Health:** Soothing for mouth and throat / Reliefs in case of irritation of throat and pharynx / Soothing and pleasant effect on throat, pharynx and vocal cords<br>Vitamin C contributes to the normal function of the immune system.

12	marshmallow, chamomile, honey	respiratory	It has a beneficial effect on dry cough complaints.	**3723 - Althaea officinalis L. (Common name: Marshmallow) - Respiratory health:** Soothing for mouth and throat / Reliefs in case of tickle in the throat and pharynx / Soothing and pleasant effect on throat, pharynx and vocal cords<br>**3312 - Chamomile (Matricaria chamomilla L.) - Respiratory health:** Release of the respiratory tract - Supportive and soothing in case of dry cough, tickle in the throat - Soothing the throat - Respiratory comfort - Helps to soften respiratory troubles like coughs and sore throat in a natural way - Helps maintain respiratory health<br>**3313 - Chamomilla recutita (L.) (Chamomile-extract) – relief of airways with common cold:** relief of airways caused by common cold.<br>**3443 - Matricaria recutita L. (Common name: Chamomile) - Respiratory health:** Soothing for mouth and throat / Reliefs in case of tickle in the throat and pharynx / Soothing and pleasant effect on throat, pharynx and vocal cords<br>**4079 - Matricaria recutita L. (Common name: Chamomile) - Respiratory health:** Soothing for mouth and throat / Reliefs in case of irritation of throat and pharynx / Soothing and pleasant effect on throat, pharynx and vocal cords
13	primrose, thyme, honey	respiratory	Helps expectoration.	**4258 - Primula veris (Common Name: Cowslip) - Health of the upper respiratory tract:** Promotes upper respiratory tract health.<br>**4259 - Primula veris L. syn. Primula officinalis L. (Common name: Cowslip) - Respiratory health:** Soothing for mouth and throat / Reliefs in case of tickle in the throat and pharynx / Soothing and pleasant effect on throat, pharynx and vocal cords<br>**4468 - Primula officinalis (promrose)-radix- - it sustains the respiratory apparatus; the saponins have a secretolytic and secretomotor action**: it favours expectoration of bronchial secretions.<br>**2149 - Thymus vulgaris/zygis (Common Name: Thyme) - Health of the upper respiratory tract:** Soothing for troat and chest /contributes to wellbeing of chest and throat /contributes to a fresh breath '-Good for respiratory tract and/or throat, -Soothens the respiratory tract<br>**2687 - Common Thyme (Thymus vulgaris, Thymus zygis) - Supports secretion of mucus in the upper respiratory tract:** Eases expectoration. Helps with dry cough.<br>**4167 - Thymus vulgaris L. (Common name: Thyme) - Respiratory health:** Soothing for mouth and throat / Reliefs in case of irritation of throat and pharynx / Soothing and pleasant effect on throat, pharynx and vocal cords
31	marshmellow, vitamin C	respiratory	Food supplement for maintaining the health of respiratory organs.<br>Marshmallow has a soothing and pleasant effect on throat, pharynx, and vocal cords.<br>It provides relief in case of throat, pharynx, and vocal cord irritation.	**3723 - Althaea officinalis L. (Common name: Marshmallow) - Respiratory health:** Soothing for mouth and throat / Reliefs in case of tickle in the throat and pharynx / Soothing and pleasant effect on throat, pharynx and vocal cords
32	marshmallow, thyme, primrose, honey	respiratory	Herbal food supplement (oral solution) for children for throat and respiratory organs.<br>Marshmallow root, thyme herb, and primrose root have a soothing effect on irritated mucous membrane of the throat and upper respiratory tract.	**3724 - Althaea officinalis L. (Common name: Marshmallow) - Respiratory health:** Soothing for mouth and throat / Reliefs in case of tickle in the throat and pharynx / Soothing and pleasant effect on throat, pharynx and vocal cords<br>**4258 - Primula veris (Common Name: Cowslip) - Health of the upper respiratory tract:** Promotes upper respiratory tract health.

				**4259 - Primula veris L. syn. Primula officinalis L. (Common name: Cowslip) - Respiratory health:** Soothing for mouth and throat / Reliefs in case of tickle in the throat and pharynx / Soothing and pleasant effect on throat, pharynx and vocal cords<br>**4468 - Primula officinalis (promrose)-radix- - it sustain the respiratory apparatus; the saponins have a secretolytic and secretomotor action**: it favours expectoration of bronchial secretions.<br>**2149 - Thymus vulgaris/zygis (Common Name : Thyme) - Health of the upper respiratory tract:** Soothing for troat and chest /contributes to wellbeing of chest and throat /contributes to a fresh breath '-Good for respiratory tract and/or throat, -Soothens the respiratory tract<br>**2687 - Common Thyme (Thymus vulgaris, Thymus zygis) - Supports secretion of mucus in the upper respiratory tract:** Eases expectoration. Helps with dry cough.<br>**4167 - Thymus vulgaris L. (Common name: Thyme) - Respiratory health:** Soothing for mouth and throat / Reliefs in case of irritation of throat and pharynx / Soothing and pleasant effect on throat, pharynx and vocal cords
33	chamomile, wild candytuft, greater celandine, liquorice, caraway, lemon balm, angelica, milk thistle, mint	GIT<br>GIT	Helps with stomach discomfort and facilitates digestion.<br>Formulated based on a blend of extracts from 9 plants that by joint action facilitate the symptoms of difficult digestion (bloating, gases, stomach cramps, feeling of fullness, nausea).	**2237 - Chamaemelum nobile - common name: Chamomile, Roman chamomile - Digestion:** / "Used to facilitate the digestion" / "Contributes to the digestive comfort" / "Helps to support the digestion" / "Contributes to support the digestion".<br>**3311 - Chamaemelum nobile - common name: Chamomile, Roman chamomile - Digestion:** "Traditionally used to facilitate the digestion" / "Used to facilitate the digestion" / "Contributes to the digestive comfort" / "Helps to support the digestion" / "Contributes to support the digestion".<br>**3928 - Matricaria recutita (Common Name : Chamomile Camomile) - Digestive health:** Helps to supports the treatment of gastro-intestinal complaints such as minor spasms epigastric distension, flatulence and belching<br>**2694 - German Chamomile (Matricaria/Chamomills recutita L.) - Spasmolytic effect:** Helps with indigestion and flatulence.<br>**2238 - Chelidonium majus - Liver health:** Contributes to liver health /maintenance of normal liver function and additionally supports the digestion and the body´s purification<br>**2370 - Sweet-root/licorice (Glycyrrhiza glabra); (Deglycrrhizinated Licorice radix (DGL);; - Gastrointestinal health:** Active substances of licorice help maintain normal function of mucous membranes in the stomach and small intestine.<br>**3769 - Glycyrrhiza glabra - common name: Licorice - Digestion:** "Traditionally used to facilitate the digestion" / "Used to facilitate the digestion" / "Contributes to the digestive comfort" / "Helps to support the digestion" / "Contributes to support the digestion".<br>**4052 - Glycyrrhiza glabra ROOT - Digestion:** Helps to maintain balance and comfort in the digestive systems of people with sensitive digestions<br>**2008 - Carum carvi (Common Name : Caraway) - Digestive health:** supports digestion and digestive functions /stimulates digestion /releives fullness and windy feelings ?contributes to the normal function of intestinal tract /helps support the digestive juice flow

				**2086 - Melissa officinalis (Common Name : Lemon Balm Balm mint) - Digestive health:** Helps to support the digestion/contributes to the normal function of intestinal tract/contributes to physical wel being<br>**3732 - Angelica archangelica - common name: Angelica, European angelica - Digestion:** "Traditionally used to facilitate the digestion" / "Used to facilitate the digestion" / "Contributes to the digestive comfort" / "Helps to support the digestion" / "Contributes to support the digestion".<br>**3733 - ANGELICA ARCHANGELICA L. - Contributes to maintain physiological gastric pH and digestive health:** Stimulates the digestion. Contributes to the functions of the intestinal tract. Helps the physiological pH balance of the stomach.<br>**4261 - Angelica archangelica (Common Name : Angelica) - Appetite:** Contributes to appetite /helps to support the digestion helps to promote appetite in cases of loss of appetite<br>**2092 - Mentha piperita (Common Name : Mint) - Intestinal and digestive health / Stomach health:** Helps to supports a healthy digestion /has a positive influence on intestinal health /contributes to digestive functions /contributes to the normal function of intestinal tract /helps keep the stomach healthy
34	marshmallow	respiratory<br>respiratory	Marshmallow has a soothing and pleasant effect on the throat, pharynx, and vocal cords.<br>It provides relief in case of throat and pharynx irritation.	**3723 - Althaea officinalis L. (Common name: Marshmallow) - Respiratory health:** Soothing for mouth and throat / Reliefs in case of tickle in the throat and pharynx / Soothing and pleasant effect on throat, pharynx and vocal cords
38	fennel, chamomile, caraway, mint	GIT<br>GIT<br>GIT	Food supplement for children with extracts of fennel fruit, chamomile flower, caraway fruit, and mint oil for improvement of digestion and elimination of the intestinal gases.<br>Fennel and caraway fruits and mint essential oil contribute to the normal function of the digestive tract.<br>Chamomile flower helps with flatulence issues.	**2237 - Chamaemelum nobile - common name: Chamomile, Roman chamomile - Digestion:** / "Used to facilitate the digestion" / "Contributes to the digestive comfort" / "Helps to support the digestion" / "Contributes to support the digestion".<br>**3311 - Chamaemelum nobile - common name: Chamomile, Roman chamomile - Digestion:** "Traditionally used to facilitate the digestion" / "Used to facilitate the digestion" / "Contributes to the digestive comfort" / "Helps to support the digestion" / "Contributes to support the digestion".<br>**3928 - Matricaria recutita (Common Name : Chamomile Camomile) - Digestive health:** Helps to supports the treatment of gastro-intestinal complaints such as minor spasms epigastric distension, flatulence and belching<br>**2694 - German Chamomile (Matricaria/Chamomills recutita L.) - Spasmolytic effect:** Helps with indigestion and flatulence.<br>**2008 - Carum carvi (Common Name : Caraway) - Digestive health:** supports digestion and digestive functions /stimulates digestion /releives fullness and windy feelings ?contributes to the normal function of intestinal tract /helps support the digestive juice flow<br>**2051 - Foeniculum vulgare ssp. Cappillaceum var. vulgare. DRIED FRUIT - Appetite, digestion & elimination:** Supports appetite, digestion and elimination. Supports the health of the digestive tract.<br>**2692 - Fennel (Foeniculum vulgare) - Spasmolytic and carminative effect:** Helps with flatulence and belly spasm.

				**2368 - Combination of; Sweet fennel seed (Foeniculum dulce), mint leaf (Mentha piperita) and chamomile flower (Matricaria chamomilla); - Digestive system:** The combination contributes to maintain normal digestion; The combination helps maintain well-being of digestive system and avoid mild digestive complaints like satiety and distension.
40	manna ash, plum, apple, cheeses, inulin, apple pectin	GIT	Food supplement with herbal extracts, fibers and fruit juices, intended for children and adults, for regulating the physiological functions of the intestinal tract.	**2258 - Fraxinus ornus - common name : Manna - Constipation / Intestinal Health:** "Support gastrointestinal health" / "Helps to support the digestion" / "Maintenance of the intestinal functions" / "Supports better bowel performance" / "Supports regular bowel movements" / "For a regular bowel motion" / "Supports bowel transit" / "Maintains a regular bowel function" / "Helps to maintain bowel function" / "Helps to maintain optimum digestive comfort" / "Helps to regulate transit time" / "Helps to maintain a good transit"
		GIT	Manna improves bowel transit and contributes to the normal function of the digestive tract.	
41	wheat germ, pollen, great yellow gentian, common centaury, fenugreek		Food supplement for children and adults with pollen and herbal extracts.	**3135 - Bee pollen - enhances appetite:** enhances appetite
		GIT	Pollen and fenugreek seed contribute to a normal appetite.	**4323 - Pollen (from multiflowers) - is effective in the management of gastrointestinal disorders:** Contributes to the gastrointestinal well-being
		GIT	Gentian root, centaury herb and fenugreek seed contribute to the normal function of the digestive tract.	**3952 - Trigonella foenum-graecum (Common Name : Fenugreek) - Appetite:** Contributes to appetite /helps to support digestion
				**3765 - Gentiana lutea - common name: Gentian - Digestion:** "Traditionally used to facilitate the digestion" / "Used to facilitate the digestion" / "Contributes to the digestive comfort" / "Helps to support the digestion" / "Contributes to support the digestion".
				**2236 - Centaurium erythraea - Digestive health:** helps to support the digestion ; contributes to the function of intestinal tract
				**2720 - Centaurium erythrea - common name: Centaury herb - Digestion:** "Traditionally used to facilitate the digestion" / "Used to facilitate the digestion" / "Contributes to the digestive comfort" / "Helps to support the digestion" / "Contributes to support the digestion".
42	chestnut, yarrow, stork's bill, yellow sweet clover, white sweet clover, pot marigold	CVS	The product is intended to maintain physiological blood flow through the venous vessels of the lower extremities and improve the health of veins and capillaries.	**3720 - Aesculus hippocastanum - common name : Horrehound, Horse chestnut - Vascular and Vein Health:** "Traditionally used for the good circulation of blood in microvessels" / "Traditionally used to decrease the sensations of heavy leggs" / "Used for the good circulation of blood in microvessels" / "Helps to decrease the sensations of heavy leggs"
			The product is not intended for the diagnosis or treatment of any disease.	**3954 - Aesculus hippocastanum (Common Name : Horse chestnut) - Vein health/ Blood circulation:** Helps maintain healthy blood circulation/promotes circulatory health//helps maintain healthy venous circulation in the legs
				**4058 - Horse chestnut (Aesculus hippocastamon) - Heart health, venous sufficiency:** Horse chestnut extracts help maintain proper circulation Horse chestnut extracts help support healthy veins and capillaries
				**4341 - Achillea millefolium-flowers-Asteraceae-Coada soricelului-Yarrow - Vascular Health due to salicylic acid:** helps to maintain normal vascular function
				**3453 - Melilotus officinalis - common name : Melilot, Sweet clover - Vascular and Vein Health:** "Traditionally used for the good circulation of blood in microvessels" / "Traditionally used to decrease the sensations of heavy leggs" / "Used for the good circulation of blood in microvessels" / "Helps to decrease the sensations of heavy leggs"

				**3868 - Melilotus officinale (Common Name : Melilot, sweet clover) - Vein health:** Contributes to circulatory health /tired legs /light legs
43	vitex		Food supplement with water-ethanol extract of vitex fruit, intended for women in reproductive period and menopause.	**3653 - VITEX AGNUS-CASTUS L. - Contributes to physical well-being:** Helps during the premenstrual cycle.Contributes to relieve the menopause symptomsHelps maintaining mobility and flexibility of joints.
		urogenital	The product contains a source of biologically active compounds significant for the general well-being of women in the days preceding the menstrual cycle and women with irregular cycles.	**3654 - VITEX AGNUS-CASTUS L. - Helps to alleviate menopausal and premenstrual symptoms:** Helps to maintain a physiological well-being during the menstrual cycle. Contributes to the female hormonal balance during clymaterium. Contributes to relieve the menopause symptoms.
		immune / urogen / nerv	Vitex contributes to the general well-being, normal menstrual cycle, alleviation of menopausal and menstrual discomfort and maintenance of normal cognitive functions.	**3655 - VITEX AGNUS-CASTUS L. - Helps to maintain good cognitive functioning:** Contributes to recover physical and mental well-being.
				**3842 - Vitex agnus-castus (Common Name : Chasteberry, Chaste fruit, Monk's pepper) - Menstruation:** Helps to maintain good comfort before and during menstrual cycle
44	Lady's mantle, yarrow, pot marigold, bigroot geranium, raspberry, parsley		Food supplement based on water-alcohol extracts of Lady's mantle herb, yarrow herb, marigold flower, geranium herb, raspberry leaf, and parsley leaf.	**4410 - Geranium robertianum, herba-Robert Geranium, herbs-250 mg/cps - Sexual health:** Helps to maintain good sexual relations.
		urogenital	Geranium herb helps to preserve good sexual functions.	**2203 - Alchemilla vulgaris - Menstruation:** Helps to maintain good comfort before and during menstrual cycle
		urogenital	Lady's mantle herb helps maintain comfort before and during the menstrual cycle.	**3717 - Achillea millefolium - common name: Yarrow, Achillea, Milfoil, Millefolium - Digestion:** "Traditionally used to facilitate the digestion" / "Used to facilitate the digestion" / "Contributes to the digestive comfort" / "Helps to support the digestion" / "Contributes to support the digestion".
		urogenital	Yarrow herb soothes abdominal cramps in women.	**2760 - Achillea millefollium - Douleurs mensyeslles:** Chez les femmes, apaise les crampes abdominales périodiques
45	mistletoe, lemon balm, yarrow, pot marigold, valerian		Food supplement based on a blend of herbal extracts.	**3651 - VISCUM ALBUM L. - Supports cardiac function:** Contributes to vascular health. Helps the microcirculation trophism, increasing the permeability and capillary microcirculation.
		CVS	Mistletoe herb contributes to the normal function of the heart.	**2085 - Melissa officinalis (Common Name : Lemon Balm) - Cognitive and mental health:** Helps maintain positive mood and good cognitive functioning/contributes to optimal relaxation/helps to support the relaxation and mental and physical wel being/contributes to a normal helps to maintain a healthy sleep
		nerv / endocrin	Lemon balm herb contributes to maintaining hormonal balance, good mood, and cognitive functioning, optimal relaxation, physical and mental well-being and preserving normal sleep.	**2086 - Melissa officinalis (Common Name : Lemon Balm Balm mint) - Digestive health:** Helps to support the digestion/contributes to the normal function of intestinal tract/contributes to physical wel being

	nervous	Marigold flower helps the body overcome stress and strain and helps in adapting to age-related changes.	**2302 - Melissa officinalis - common name : melissa, lemon balm, balm mint, sweet balm, common balm - Relaxing effect - helps to find sleep:** "Used to help to find a better sleep" / "Used to decrease tenseness" / "Used to decrease restleness" / "Used to decrease irritability" / "Helps to find a better sleep" / "Contributes to find a better sleep" / "Helps to decrease tenseness" / "Contributes to decrease tenseness" / "Helps to decrease restleness" / "Contributes to decrease restleness" / "Helps to derease irritability" / "Contributes to decrease irritability".
	nervous	Valerian root contributes to maintaining mental well-being.	**4444 - Melissa officinalis, herba, Common balm, herbs - Glandular System Health:** Maintain the natural hormonal balance of the human body<br><br>**2084 - Melissa extract [Dry extract from leaves of Melissa officinalis L., drug/native extract ratio (4 - 6) : 1, solvent of extraction Methanol/Water , min 1.8% rosmarinic acid] - For cognitive and mental health:** *Clinically/scientifically proven to help maintaining positive mood and good cognitive functioning, Contributes to optimal relaxation, Helps to support the relaxation and mental and physical well-being, Contributes to a good and calm rest<br><br>**2301 - Melissa extract [Dry extract from leaves of Melissa officinalis L., drug/native extract ratio (4 - 6) : 1, solvent of extraction Methanol/Water , min 1.8% rosmarinic acid] - For insomnia and mental health:** Support calmness and to help sleep onset Celp normalise/ promote sleep (onset). Contributes to optimal relaxation, Contributes to a normal/ helps to maintain a healthy sleep. Contributes to a good and calm rest.<br><br>**2405 - Calendula officinalis (Marigold) - Healing processes, invigoration the body:** 1. Contains herbs with allergy reducing, anti-inflammatory properties 2. Helps the body overcome stress and exertion, prevents setting in of changes associated with age<br><br>**2349 - Valerian extract [Dry extract from roots of Valeriana officinalis L., drug/native extract ratio (3 - 6) : 1, solvent of extraction Ethanol/Water, min 0.3% valerenic acid] - For mental health:** To support calmness and in case of irritability. Helps you to cope calmly with the stress of a busy lifestyle. Support of mental well-being in cases of tension and stress. Contributes to optimal relaxation. Helps to support the relaxation<br><br>**3837 - VALERIANA OFFICINALIS L. - Helps to maintain good cognitive functioning:** Contributes to recover physical and mental well-being.<br>**4222 - Valeriana officinalis (Common Name : Valerian) - Mental health:** Helps to maintain a natural sleep/helps maintain normal quality of sleep/helps you cope calmly with the stress of a busy lifestyle Support of mental welbeing in cases of tension and stress/contributes to optimal relaxation/helps to support the relaxation and mental and physical well being To help sleep onset, Clinically/scientifically proven to help normalise/promote sleep (onset), Valerian helps to maintain a natural sleep, To support calmness and in case of irritability Helps you cope calmly with the stress of a busy lifestyle, Support of mental well-being in cases of tension and stress, Contributes to optimal relaxation, Helps to support the relaxation and mental and physical well-being

46	echinacea	immune	Food supplement intended for immunity.	**4257 - Echinacea angustifolia/pallida (Common Name : Echinacea, pale coneflower) - Immune health:** Support of the body´s defence /contributes to the /supports the immune system
			Alcohol-free oral drops; liquid food supplement with purple echinacea extract.	**2366 - Purple;coneflower;(Echinacea purpurea);; - Immune system health:** Contribute to the proper function of the body´s defensive system;Echinacea purpurea helps the function of the natural defensive system;
		immune	Echinacea contains biologically active components intended for immunity.	
49	bigroot geranium, elder, acerola, dog rose, zinc, vitamin D, copper	respiratory	The syrup is a food for special medical purposes intended for the nutritional needs of individuals in conditions of cold and flu, cough, sore throat and inflammation of the mucous membrane of the upper respiratory tract, in children older than 3 years.	**3872 - Pelargonium reniforme/sidoids (Common Name : Geranium) - Respiratory health:** -soothens the respiratory tract '- Soothing for throat - supportive and soothing in case of tickle in the throat - to use in case of temporary croakiness
			The product contains extracts of: geranium root, black elder fruit, rose hip fruit which contains vitamin C, acerola fruit which contains primarily vitamin C, vitamins A, B group and D, carotenoids, bioflavonoids, zinc, and copper.	**4090 - Pelargonium reniforme/sidoids (Common Name : Geranium) - Respiratory health:** Respiratory comfort/helps to soften respiratory troubles like coughs, sore throats in a natural way
				**2134 - Sambucus nigra (Common Name : Elder) - Immune health:** Support of the body´s defence /Helps to supports the immune system<br>**3680 - Rosa canina (Common Name : Rose Hip) - Respiratory health:** helps to soothe common cold/contributes to physical wel being/contributes to the body´s defences<br>**3682 - Rosa canina (Common Name : Rose Hip) - Immune health:** Contributes to the resistance against health precarious microorganism/contributes to physical wel-being<br>**3881 - Rosa canina (Common Name : Dog rose / Rose hip) - Respiratory health:** /contributes to the body´s defences
57	elder, beta glucan, bigroot geranium		Food supplement representing a combination of elder fruit extract, African geranium root and beta-glucan from yeast. It is recommended especially during the autumn and winter period.	**3872 - Pelargonium reniforme/sidoids (Common Name : Geranium) - Respiratory health:** -soothens the respiratory tract '- Soothing for throat - supportive and soothing in case of tickle in the throat - to use in case of temporary croakiness
		immune	Bioactive ingredients of elder fruit contribute to the normal function of the immune system.	**4090 - Pelargonium reniforme/sidoids (Common Name : Geranium) - Respiratory health:** Respiratory comfort/helps to soften respiratory troubles like coughs, sore throats in a natural way
		respiratory	The root of African geranium has a beneficial effect on the mucous membrane of the throat and respiratory tract.	**2134 - Sambucus nigra (Common Name: Elder) - Immune health:** Support of the body´s defence /Helps to supports the immune system
62	echinacea, dog rose, zinc, copper	immune	Helps the preservation of the normal function of the immune system of the organism.	**4257 - Echinacea angustifolia/pallida (Common Name : Echinacea, pale coneflower) - Immune health:** Support of the body´s defence /contributes to the /supports the immune system<br>**2366 - Purple;coneflower;(Echinacea purpurea);; - Immune system health:** Contribute to the proper function of the body´s defensive system;Echinacea purpurea helps the function of the natural defensive system;<br>**3682 - Rosa canina (Common Name : Rose Hip) - Immune health:** Contributes to the resistance against health precarious microorganism/contributes to physical wel-being

67	fennel, alder buckthorn, field horstail, birch, ginger		Food supplement based on extracts of fennel fruit, buckthorn bark, field horstail herb, birch leaf and ginger rhizome.	**2051 - Foeniculum vulgare ssp. Cappillaceum var. vulgare. DRIED FRUIT - Appetite, digestion & elimination:** Supports appetite, digestion and elimination. Supports the health of the digestive tract.
		GIT	Fennel fruit and buckthorn bark can help regulate intestinal passage and eliminate digestive gases.	**3875 - Rhamnus frangula (Common Name : Buckthorn) - Intestinal health:** Contributes to soft stools /supports bowel movement /contributes to the working of the intestines and regular bowel movement.
		GIT	Field horstail herb may help in protecting the liver and stimulating bile secretion.	**4316 - Equisetum arvense (horsetail) green and sterile areal stems - Hepatic system: has a protective effect on hepatocyts:** improves liver function thanks to its protective effect/is rich of protective compounds that protect liver cells against toxic substances
		GIT	Birch leaf may have a positive effect on cholesterol metabolism and digestive tract health.	**4366 - Betula pendula, folium, European White Birch, leafs - Blood cholesterol:** Supports the normal level of blood cholesterol.
		immune	Ginger rhizome may help increase tone and vitality of the body.	**2402 - Betula pendula (Birch) - Digestive health:** 1. Contains herbs that have liver, kidney and intestinal tract activity promoting properties 2. Beneficially affects digestion and promotes body's detoxification processes
				**2172 - Zingiber officinale (Common Name : Ginger) - Digestive health:** Helps to support the digestion/contributes to the normal function of intestinal tract/contributes to physical well being/contributes to the normal functioning of the stomach in case of early pregnancy
				**2726 - Ginger (Zingiber officinale) - Tonus/vitality:** Contributes to enhancement of vitality/energy. May help energy enhancement. Useful in case of fatigue. Have stimulating and tonic properties that contribute to the resistance against fatigue.
71	propolis, ginseng	immune	The product is intended to strengthen immunity and overall well-being.	No defined "on hold" claims for propolis
			The product is not a medicine nor a diagnostic device.	**3672 - Panax ginseng - Immune health:** Ginseng contributes to the natural defences and proper fuctioning of the immune system
				**3674 - Panax Ginseng - Vitality:** Helps to promote vitality
				**3930 - Panax ginseng (Common name: Ginseng) - Ginseng and immunity:** Ginseng supports the immune system
				**2672 - Ginseng, extract from root - Effective substances, ginsenosides:** Strengthening the human body, supply of lacking energy and positive life force. Antioxidant.
76	marshmallow, rose hip	respiratory	Food supplement intended for the respiratory organs and mucous membranes of the mouth and throat.	**3723 - Althaea officinalis L. (Common name: Marshmallow) - Respiratory health:** Soothing for mouth and throat / Reliefs in case of tickle in the throat and pharynx / Soothing and pleasant effect on throat, pharynx and vocal cords
		respiratory	Marshmallow soothes irritation of the mucous membrane of the oral cavity and throat and alleviates dry irritative cough.	**3680 - Rosa canina (Common Name : Rose Hip) - Respiratory health:** helps to soothe common cold/contributes to physical wel being/contributes to the body´s defences
79	marshmallow, primrose, chamomile, basil, vitamin C	respiratory	Helps with cough.	**3723 - Althaea officinalis L. (Common name: Marshmallow) - Respiratory health:** Soothing for mouth and throat / Reliefs in case of tickle in the throat and pharynx / Soothing and pleasant effect on throat, pharynx and vocal cords

		respiratory	Food supplement containing herbal extracts of marshmallow root, primrose root, chamomile flower and basil leaf, which contribute to maintaining normal function of the mucous membranes of the respiratory tract.	**4258 - Primula veris (Common Name: Cowslip) - Health of the upper respiratory tract:** Promotes upper respiratory tract health.
		respiratory	The product is intended for individuals with symptoms of cold and flu accompanied by cough.	**4259 - Primula veris L. syn. Primula officinalis L. (Common name: Cowslip) - Respiratory health:** Soothing for mouth and throat / Reliefs in case of tickle in the throat and pharynx / Soothing and pleasant effect on throat, pharynx and vocal cords
				**4468 - Primula officinalis (promrose)-radix- - it sustain the respiratory apparatus; the saponins have a secretolytic and secretomotor action**: it favours expectoration of bronchial secretions.
				**4450 - Ocimum basilicum-herb-fruits, flowers, leaf/basil - protects lung cells:** Helps to soothe common cold/pleasant for cough and croakiness/contributes to physical well-being
				**3312 - Chamomile (Matricaria chamomilla L.) - Respiratory health:** Release of the respiratory tract - Supportive and soothing in case of dry cough, tickle in the throat - Soothing the throat - Respiratory comfort - Helps to soften respiratory troubles like coughs and sore throat in a natural way - Helps maintain respiratory health
				**3313 - Chamomilla recutita (L.) (Chamomile-extract) – relief of airways with common cold:** relief of airways caused by common cold.
				**3443 - Matricaria recutita L. (Common name: Chamomile) - Respiratory health:** Soothing for mouth and throat / Reliefs in case of tickle in the throat and pharynx / Soothing and pleasant effect on throat, pharynx and vocal cords
				**4079 - Matricaria recutita L. (Common name: Chamomile) - Respiratory health:** Soothing for mouth and throat / Reliefs in case of irritation of throat and pharynx / Soothing and pleasant effect on throat, pharynx and vocal cords
81	hawthorn		Herbal drops based on extract of hawthorn leaf with flower.	**2250 - Crataegus laevigata - common name : Hawthorn - Relaxing effect - Helps to find sleep:** / "Used to help to find a better sleep" / "Used to decrease tenseness" / "Used to decrease restleness" / "Used to decrease irritability" / "Helps to find a better sleep" / "Contributes to find a better sleep" / "Helps to decrease tenseness" / "Contributes to decrease tenseness" / "Helps to decrease restleness" / "Contributes to decrease restleness" / "Helps to derease irritability" / "Contributes to decrease irritability".
		CVS	The active ingredients of hawthorn leaf with flower support normal heart activity, contribute to reducing tension and facilitate the onset of sleep.	**3747 - Crataegus monoggyna (Common Name : Hawthorn) - Heart health / Vascular system:** Supports circulation, relaxing, calming
		nervous		**2789 - Crataegus laevigata Hawthron extract - increase oxygen inflow and improves peripheral blood circulation:** Hawthron extract supports heart functions, increases oxygen inflow and improves peripheral blood circulation
82	nettle	musculo-skeletal	Herbal drops based on extract of nettle leaf.	**4497 - Urtica dioica (stinging nettele)-herba- - it sustain the osteoarticulary system; the caffeoylmalic acids has an antiinflamatory effect:** it contains minerals (iron) and reduces anaemia. /

		immune	The active ingredients of nettle leaf act favorably in cases of anemia and support the normal function of the musculoskeletal and immune system.	
84	valerian		Herbal drops based on extract of valerian root.	**2500 - Valeriana officinalis (Valerian) - Cardiovascular health:** 1. For cardiovascular health 2. Improves function of the heart 3. Strengthens blood vessels 4. Increases elasticity and strength of blood vessel and capillary walls 5. Normalizes blood pressure
		nerv / CVS / GIT	The active ingredients of valerian root act favorably in cases of stress and insomnia and support the normal function of the cardiovascular and digestive system.	**2501 - Valeriana officinalis (Valerian) - Digestive health:** 1. Reduces digestive system disorders and spasms 2. Promotes the activity of the digestive system
				**3837 - VALERIANA OFFICINALIS L. - Helps to maintain good cognitive functioning:** Contributes to recover physical and mental well-being.
				**4222 - Valeriana officinalis (Common Name : Valerian) - Mental health:** Helps to maintain a natural sleep/helps maintain normal quality of sleep/helps you cope calmly with the stress of a busy lifestyle Support of mental welbeing in cases of tension and stress/contributes to optimal relaxation/helps to support the relaxation and mental and physical well being To help sleep onset, Clinically/scientifically proven to help normalise/promote sleep (onset), Valerian helps to maintain a natural sleep, To support calmness and in case of irritability Helps you cope calmly with the stress of a busy lifestyle, Support of mental well-being in cases of tension and stress, Contributes to optimal relaxation, Helps to support the relaxation and mental and physical well-being

Table S5. Labels of herbal food supplements which carried claims partially non-compliant with the List of authorized health claims and ′on-hold′ claims from the EFSA Register of questions.

N°	Composition	Purpose/ effect on the organ system	Claims from the labels of herbal food supplements	′on hold′ health claims / authorised health claims
6	rose hip, primrose	respiratory	Traditionally used as an expectorant and support in the **treatment** of productive cough.	**4258 - Primula veris (Common Name: Cowslip) - Health of the upper respiratory tract:** Promotes upper respiratory tract health.
			The active ingredients of primrose (saponins) locally irritate the mucous membrane of the respiratory organs, thereby increasing the secretion of bronchial mucus.	**4259 - Primula veris L. syn. Primula officinalis L. (Common name: Cowslip) - Respiratory health:** Soothing for mouth and throat / Reliefs in case of tickle in the throat and pharynx / Soothing and pleasant effect on throat, pharynx and vocal cords
			Saponins reduce the surface tension of mucus, leading to a decrease in the density and viscosity of secretions, thus facilitating expectoration.	**4468 - Primula officinalis (promrose)-radix- - it sustain the respiratory apparatus; the saponins have a secretolytic and secretomotor action**: it favours expectoration of bronchial secretions.
			Primrose, through β2 receptors located in the wall of the respiratory organs, dilates the bronchi and facilitates breathing.	**3680 - Rosa canina (Common Name: Rose Hip) - Respiratory health:** helps to soothe common cold/contributes to physical wel being/contributes to the body´s defences
		antimicrobial / anti-inflammatory	Primrose exhibits antimicrobial and anti-inflammatory effects.	
7	rose hip, plantago	respiratory	Helps maintaining the normal functioning of the respiratory mucous membranes, in cases of irritation of the mucous membranes of the mouth and throat accompanied by dry cough.	**3510 - PLANTAGO LANCEOLATA L. - Contributes to maintain a normal intestinal function:** Stimulates the growth of beneficial intestinal micro flora.
		respiratory	Plantago is traditionally used to reduce inflammation and symptoms of colds and flu.	**3512 - PLANTAGO LANCEOLATA L. - Helps to maintain a healthy intestinal microbial balance:** Stimulates the growth of beneficial intestinal micro flora.
		antimicrobial	The active components of plantago have antimicrobial effect.	**3791 - Plantago lanceolata L. (Common name: Ribwort plantain) - Respiratory health:** Soothing for mouth and throat / Reliefs in case of tickle in the throat and pharynx / Soothing and pleasant effect on throat, pharynx and vocal cords
				**3680 - Rosa canina (Common Name : Rose Hip) - Respiratory health:** helps to soothe common cold/contributes to physical wel being/contributes to the body´s defences
8	marshmallow, chamomile, rose hip, basil	respiratory	Helps with cough.	**3723 - Althaea officinalis L. (Common name: Marshmallow) - Respiratory health:** Soothing for mouth and throat / Reliefs in case of tickle in the throat and pharynx / Soothing and pleasant effect on throat, pharynx and vocal cords
		respiratory	The product is intended for children with symptoms of cold, flu and similar conditions, accompanied by cough.	**3312 - Chamomile (Matricaria chamomilla L.) - Respiratory health:** Release of the respiratory tract - Supportive and soothing in case of dry cough, tickle in the throat - Soothing the throat - Respiratory comfort - Helps to soften respiratory troubles like coughs and sore throat in a natural way - Helps maintain respiratory health

		respiratory	The product combines herbal extracts that, thanks to their components (mucilage, essential oils, saponosides), have positive effect on the mucous membranes of the respiratory tract.	**3313 - Chamomilla recutita (L.) (Chamomile-extract) – relief of airways with common cold:** relief of airways caused by common cold.
		antioxidative	Honey contributes to the respiratory tract health due to the presence of phytochemicals with antioxidant properties.	**3443 - Matricaria recutita L. (Common name: Chamomile) - Respiratory health:** Soothing for mouth and throat / Reliefs in case of tickle in the throat and pharynx / Soothing and pleasant effect on throat, pharynx and vocal cords<br>**4079 - Matricaria recutita L. (Common name: Chamomile) - Respiratory health:** Soothing for mouth and throat / Reliefs in case of irritation of throat and pharynx / Soothing and pleasant effect on throat, pharynx and vocal cords<br>**3680 - Rosa canina (Common Name : Rose Hip) - Respiratory health**: helps to soothe common cold/contributes to physical wel being/contributes to the body´s defences<br>**4450 - Ocimum basilicum-herb-fruits, flowers, leaf/basil - protects lung cells:** Helps to soothe common cold/pleasant for cough and croakiness/contributes to physical well-being
9	marshmallow, chamomile, vitamin C	respiratory	The extract of marshmallow root contains a significant amount of mucus that covers the mucous membranes in a thin layer, thus protecting them from irritation.	**3723 - Althaea officinalis L. (Common name: Marshmallow) - Respiratory health:** Soothing for mouth and throat / Reliefs in case of tickle in the throat and pharynx / Soothing and pleasant effect on throat, pharynx and vocal cords
		respiratory	Because of this property, it is used in respiratory tract disorders (to soothe dry, irritative and productive cough), as well as in the irritation of mucous membranes of digestive organs.	**3312 - Chamomile (Matricaria chamomilla L.) - Respiratory health:** Release of the respiratory tract - Supportive and soothing in case of dry cough, tickle in the throat - Soothing the throat - Respiratory comfort - Helps to soften respiratory troubles like coughs and sore throat in a natural way - Helps maintain respiratory health
		antiseptic	The active principles of chamomile flower extract possess antiseptic and spasmolitic effect, thus in combination with marshmallow and vitamin C, they form a completely natural preparation that has beneficial effect on the respiratory and digestive organs.	**3313 - Chamomilla recutita (L.) (Chamomile-extract) – relief of airways with common cold:** relief of airways caused by common cold.
		immune	The addition of vitamin C enhances **the prevention** of various infections. It increases the phagocytic power of blood cells - leukocytes, thus enhancing the body's resistance to microorganisms. It ranks among the most effective antioxidants.	**3443 - Matricaria recutita L. (Common name: Chamomile) - Respiratory health:** Soothing for mouth and throat / Reliefs in case of tickle in the throat and pharynx / Soothing and pleasant effect on throat, pharynx and vocal cords
		antioxidative		**4079 - Matricaria recutita L. (Common name: Chamomile) - Respiratory health:** Soothing for mouth and throat / Reliefs in case of irritation of throat and pharynx / Soothing and pleasant effect on throat, pharynx and vocal cords
10	primrose, thyme, vitamin C	respiratory	Saponins found in primrose root facilitate expectoration. They promote the excretion of mucus from the bronchi, dilute the dense secretions and facilitate expectoration by gently stimulating the vagal endings in the gastric mucous membrane and stimulating bronchial gland secretion.	**4258 - Primula veris (Common Name: Cowslip) - Health of the upper respiratory tract:** Promotes upper respiratory tract health.
		antiseptic	Thymol, found in thyme, has a mild antiseptic effect.	**4259 - Primula veris L. syn. Primula officinalis L. (Common name: Cowslip) - Respiratory health:** Soothing for mouth and throat / Reliefs in case of tickle in the throat and pharynx / Soothing and pleasant effect on throat, pharynx and vocal cords

		respiratory	The active principles from the extracts of these plants exhibit expectorant, bronchospasmolytic and antiseptic effects.	**4468 - Primula officinalis (promrose)-radix- - it sustain the respiratory apparatus; the saponins have a secretolytic and secretomotor action**: it favours expectoration of bronchial secretions.
		immune / antioxidative	The addition of vitamin C enhances **the prevention** of various infections. It increases the phagocytic power of blood cells - leukocytes, thus enhancing the body's resistance to microorganisms. It ranks among the most effective antioxidants.	**2149 - Thymus vulgaris/zygis (Common Name : Thyme) - Health of the upper respiratory tract:** Soothing for troat and chest /contributes to wellbeing of chest and throat /contributes to a fresh breath '-Good for respiratory tract and/or throat, - Soothens the respiratory tract
		respiratory	Menthol has a mild local anesthetic effect, inducing a pleasant cooling sensation that eases breathing and alleviates cold symptoms.	**2687 - Common Thyme (Thymus vulgaris, Thymus zygis) - Supports secretion of mucus in the upper respiratory tract:** Eases expectoration. Helps with dry cough.
		respiratory	Sodium benzoate complements the expectorant effect of saponins because, through partial secretion via bronchial glands, it has a soothing effect, making mucus less viscous and easier to expectorate.	**4167 - Thymus vulgaris L. (Common name: Thyme) - Respiratory health:** Soothing for mouth and throat / Reliefs in case of irritation of throat and pharynx / Soothing and pleasant effect on throat, pharynx and vocal cords
17	blueberry, acerola, rose hip, zinc, probiotics	immune	Liquid food supplement, oral solution for the imunity.	**3706 - VACCINIUM MYRTILLUS L. - Antioxidant:** Antioxidants can protect from free radicals and helps in case of foods intake deficiency or increased amount of nutrients.
		immune	Liquid food supplement with acerola, blueberry juice, rose hip extract, zinc and tindalized probiotic bacteria intended for immunity.	**3682 - Rosa canina (Common Name : Rose Hip) - Immune health:** Contributes to the resistance against health precarious microorganism/contributes to physical wel-being
			Rose hip and acerola represent natural sources of vitamin C.	
		antioxidative	Concentrated blueberry juice contains compounds with antioxidant activity.	
		immune	Zinc contributes to the normal function of the immune system, protection of cells from oxidative stress, maintenance of normal bones, normal protein synthesis, normal cognitive function and maintenance of normal vision.	Zinc contributes to the normal function of the immune system. Zinc contributes to the protection of cells from oxidative stress. Zinc contributes to the maintenance of normal bones. Zinc contributes to normal protein synthesis. Zinc contributes to normal cognitive function. Zinc contributes to the maintenance of normal vision.
18	baobab, elder, acerola, vitamin C, zinc, copper, vitamin D	immune	The syrup is a food supplement which contains baobab fruit pulp, elder fruit, acerola fruit, zinc, copper, vitamin C and D, intended for immuniy.	**2134 - Sambucus nigra (Common Name: Elder) - Immune health:** Support of the body´s defence /Helps to supports the immune system
			Baobab fruit is a food rich in vitamins, minerals and fibers.	
		immune	Elder, vitamins C and D, zinc and copper contribute to the normal function of the immune system.	Vitamin C contributes to the normal function of the immune system. Vitamin D contributes to the normal function of the immune system. Zinc contributes to the normal function of the immune system. Copper contributes to the normal function of the immune system.
		immune	Vitamin C and copper contributes to normal energy-yielding metabolism and reduction of tiredness and fatigue.	Vitamin C contributes to normal energy-yielding metabolism. Copper contributes to normal energy-yielding metabolism. Vitamin C contributes to the reduction of tiredness and fatigue. Copper contributes to the reduction of tiredness and fatigue.

		immune<br>immune	Vitamin C, zinc and copper contribute to the protection of cells from oxidative stress.<br>Vitamin D contributes to the normal function of the immune system of children.	Vitamin C contributes to the protection of cells from oxidative stress. Zinc contributes to the protection of cells from oxidative stress. Copper contributes to the protection of cells from oxidative stress.<br>Vitamin D contributes to the normal function of the immune system of children.
19	astragalus, elder, acerola, zinc, copper, vitamin D	immune	The syrup is a food supplement which contains astragalus root extract, elder fruit, acerola fruit, zinc, copper and vitamin D, intended for immuniy.	**3259 - ASTRAGALUS MEMBRANACEUS BUNG. - Contributes to physical well-being:** Helps maintaining mobility and flexibility of joints. Contribute to the resistance during the premenstrual cycle. Contributes to relieve the menopause symptoms.
		immune	Astragalus contributes to the health of the immune system, overall physical condition and protects the body from external agents.	**3735 - Astragalus membranaceus (Common Name : Milk-vetch) - Immune health:** Supports the natural defences
		respiratory	Astragalus root contributes to the health of the respiratory tract.	**3736 - ASTRAGALUS MEMBRANACEUS BUNG. - Contributes to body defences against external agents:** Increases the physiological resistance of the organism in case of severe ambiance conditions.
		immune	Elder, copper, zinc, vitamin C and vitamin D contribute to the normal function of the immune system of children.	**3968 - Astragalus membranaceus (Common Name : Milk vetch) - Immune health:** Supports the natural defences
		immune	Vitamin C, copper and zinc contribute to the protection of cells from oxidative stress.	**2134 - Sambucus nigra (Common Name: Elder) - Immune health:** Support of the body´s defence /Helps to supports the immune system
		immune	Vitamin C contributes to normal energy-yielding metabolism and reduction of tiredness and fatigue.	Copper contributes to the normal function of the immune system. Zinc contributes to the normal function of the immune system. Vitamin C contributes to the normal function of the immune system. Vitamin D contributes to the normal function of the immune system.<br>Vitamin C contributes to the protection of cells from oxidative stress. Copper contributes to the protection of cells from oxidative stress. Zinc contributes to the protection of cells from oxidative stress.<br>Vitamin C contributes to normal energy-yielding metabolism. Vitamin C contributes to the reduction of tiredness and fatigue.
21	plantago, chamomile, basil, coltsfoot	respiratory	Helps in acute and chronic inflammation of the respiratory tract accompanied by the production of thick secretions and difficult expectoration.	**3791 - Plantago lanceolata L. (Common name: Ribwort plantain) - Respiratory health:** Soothing for mouth and throat / Reliefs in case of tickle in the throat and pharynx / Soothing and pleasant effect on throat, pharynx and vocal cords
		respiratory	Soothes irritated mucous membranes of the respiratory tract.	**3312 - Chamomile (Matricaria chamomilla L.) - Respiratory health:** Release of the respiratory tract - Supportive and soothing in case of dry cough, tickle in the throat - Soothing the throat - Respiratory comfort - Helps to soften respiratory troubles like coughs and sore throat in a natural way - Helps maintain respiratory health
		respiratory	Has a beneficial effect on dissolving mucus and successful excretion of mucus and secretions.	**3313 - Chamomilla recutita (L.) (Chamomile-extract) – relief of airways with common cold:** relief of airways caused by common cold.<br>**3443 - Matricaria recutita L. (Common name: Chamomile) - Respiratory health:** Soothing for mouth and throat / Reliefs in case of tickle in the throat and pharynx / Soothing and pleasant effect on throat, pharynx and vocal cords

				**4079 - Matricaria recutita L. (Common name: Chamomile) - Respiratory health:** Soothing for mouth and throat / Reliefs in case of irritation of throat and pharynx / Soothing and pleasant effect on throat, pharynx and vocal cords<br>**4450 - Ocimum basilicum-herb-fruits, flowers, leaf/basil - protects lung cells:** Helps to soothe common cold/pleasant for cough and croakiness/contributes to physical well-being
22	lemon balm, hops, St John's-wort, valerian, rosemary	nervous	Helps with irritability, mild hypertension, insomnia, concentration disorders, tension, anxiety and restlessness.	**2085 - Melissa officinalis (Common Name : Lemon Balm) - Cognitive and mental health:** Helps maintain positive mood and good cognitive functioning/contributes to optimal relaxation/helps to support the relaxation and mental and physical wel being/contributes to a normal helps to maintain a healthy sleep
		GIT	Has a favorable effect on stomach discomfort caused by nervousness.	**2086 - Melissa officinalis (Common Name : Lemon Balm Balm mint) - Digestive health:** Helps to support the digestion/contributes to the normal function of intestinal tract/contributes to physical wel being
		nervous	Does not cause addiction.	**2302 - Melissa officinalis - common name : melissa, lemon balm, balm mint, sweet balm, common balm - Relaxing effect - helps to find sleep:** "Used to help to find a better sleep" / "Used to decrease tenseness" / "Used to decrease restleness" / "Used to decrease irritability" / "Helps to find a better sleep" / "Contributes to find a better sleep" / "Helps to decrease tenseness" / "Contributes to decrease tenseness" / "Helps to decrease restleness" / "Contributes to decrease restleness" / "Helps to derease irritability" / "Contributes to decrease irritability".
				**2680 - Valerian-hops combination (Humulus lupulus, Valeriana officinalis) - Sleep:** The effective substances contained in combination valerian-hops affect the receptors in the brain where they are responsible for sleep and calming down nervous activity in a natural way /contributes to a healthy sleep/helps to fall asleep<br>**3710 - Humulus lupulus (Hops) - Soothing effect, effect of promoting good sleep.**<br>**3857 - Humulus lupulus (Common Name : Hops) - Digestive health:** helps to support the digestion; contributes to the function of intestinal tract<br>**4419 - Humulus lupulus (hop)-strobuli- - it sustain the central nervous system by calming down effect of humulonelor and lupulonelor:** soothing effect for the nervous system.<br>**3596 - St John's wort, Hypericum (Hypericum perforatum L.) - Psychological state / mood:** St John's wort / Hypericum helps to maintain emotional balance / balanced mood / positive mood<br>**3860 - Hypericum perforatum (Common Name : St. John's Wort) - Mental health:** Contributes to emotional balance /contributes to optimal relaxation /helps to support the relaxation /helps to maintain a healthy sleep /helps maintain a positive mood<br>**4065 - Hypericum perforatum (Common Name : St. John's Wort) - Mental health:** Contributes to emotional balance and general welbeing/contributes to optimal relaxation/helps to support the relaxation and mental and physical wel being/helps to maintain a healthy sleep/helps maintain a positive mood

				**4421 - Hypericum perforatum-Herba hyperici plant-St. John Wort - Atenuates sleep disturbances due to hypericin (a napthodianthrone):** Helps in sleep disturbances.<br>**3837 - VALERIANA OFFICINALIS L. - Helps to maintain good cognitive functioning:** Contributes to recover physical and mental well-being.<br>**4222 - Valeriana officinalis (Common Name : Valerian) - Mental health:** Helps to maintain a natural sleep/helps maintain normal quality of sleep/helps you cope calmly with the stress of a busy lifestyle Support of mental welbeing in cases of tension and stress/contributes to optimal relaxation/helps to support the relaxation and mental and physical well being To help sleep onset, Clinically/scientifically proven to help normalise/promote sleep (onset), Valerian helps to maintain a natural sleep, To support calmness and in case of irritability Helps you cope calmly with the stress of a busy lifestyle, Support of mental well-being in cases of tension and stress, Contributes to optimal relaxation, Helps to support the relaxation and mental and physical well-being<br>**2500 - Valeriana officinalis (Valerian) - Cardiovascular health:** 1. For cardiovascular health 2. Improves function of the heart 3. Strengthens blood vessels 4. Increases elasticity and strength of blood vessel and capillary walls 5. Normalizes blood pressure<br>**3563 - Rosmarinus officinalis - common name : Rosemary - Digestion:** "Traditionally used to facilitate the digestion" / "Used to facilitate the digestion" / "Helps to support normal liver function" / "Contributes to the stimulation of the production of the digestive body fluids" / "Supports the liver and biliary function" / "Contributes to the digestive comfort" / "Helps to facilitate fat digestion" / "Contributes to better fat digestion" / "Helps to support the digestion" / "Contributes to support the digestion".
23	St John's-wort, mint, lemon balm, Breckland thyme, basil, rosemary, pot marigold	nervous	Relieves the feeling of anxiety, drop in concentration, stabilize sudden mood changes and reduce the feeling of unease and fear.	**2085 - Melissa officinalis (Common Name : Lemon Balm) - Cognitive and mental health:** Helps maintain positive mood and good cognitive functioning/contributes to optimal relaxation/helps to support the relaxation and mental and physical wel being/contributes to a normal helps to maintain a healthy sleep
		nervous	Have a beneficiall effect on reducing depressive state of the organism.	**2302 - Melissa officinalis - common name : melissa, lemon balm, balm mint, sweet balm, common balm - Relaxing effect - helps to find sleep:** "Used to help to find a better sleep" / "Used to decrease tenseness" / "Used to decrease restleness" / "Used to decrease irritability" / "Helps to find a better sleep" / "Contributes to find a better sleep" / "Helps to decrease tenseness" / "Contributes to decrease tenseness" / "Helps to decrease restleness" / "Contributes to decrease restleness" / "Helps to derease irritability" / "Contributes to decrease irritability".<br>**3596 - St John's wort, Hypericum (Hypericum perforatum L.) - Psychological state / mood:** St John's wort / Hypericum helps to maintain emotional balance / balanced mood / positive mood

				**3860 - Hypericum perforatum (Common Name : St. John's Wort) - Mental health:** Contributes to emotional balance /contributes to optimal relaxation /helps to support the relaxation /helps to maintain a healthy sleep /helps maintain a positive mood<br>**4065 - Hypericum perforatum (Common Name : St. John's Wort) - Mental health:** Contributes to emotional balance and general welbeing/contributes to optimal relaxation/helps to support the relaxation and mental and physical wel being/helps to maintain a healthy sleep/helps maintain a positive mood<br>**4421 - Hypericum perforatum-Herba hyperici plant-St. John Wort - Atenuates sleep disturbances due to hypericin (a napthodianthrone):** Helps in sleep disturbances.<br>**2769 - Basilic : Ocimum Basilicum - Relaxation:** Bien être, Apaisant, Aide à maintenir un bon sommeil<br>**2094 - Mentha piperita (Common Name : Mint) - Relaxation:** Contributes to optimal relaxation /helps to support the relaxation /contributes to a normal helps to maintain a healthy sleep
24	valerian, lemon balm, hawthorn, lavender, St John's-wort, basil	endocrine	Represents a complex of herbal components that help with regulating metabolism and hormonal balance.	**2085 - Melissa officinalis (Common Name : Lemon Balm) - Cognitive and mental health:** Helps maintain positive mood and good cognitive functioning/contributes to optimal relaxation/helps to support the relaxation and mental and physical wel being/contributes to a normal helps to maintain a healthy sleep
		nervous	Reduces constant tension and hyperactivity, providing a relaxing effect.	**2302 - Melissa officinalis - common name : melissa, lemon balm, balm mint, sweet balm, common balm - Relaxing effect - helps to find sleep:** "Used to help to find a better sleep" / "Used to decrease tenseness" / "Used to decrease restleness" / "Used to decrease irritability" / "Helps to find a better sleep" / "Contributes to find a better sleep" / "Helps to decrease tenseness" / "Contributes to decrease tenseness" / "Helps to decrease restleness" / "Contributes to decrease restleness" / "Helps to derease irritability" / "Contributes to decrease irritability".
		GIT	Calm a nervous stomach.	**2769 - Basilic : Ocimum Basilicum - Relaxation:** Bien être, Apaisant, Aide à maintenir un bon sommeil
		nervous	Does not disrupt alertness and usual daily activities.	**3596 - St John's wort, Hypericum (Hypericum perforatum L.) - Psychological state / mood:** St John's wort / Hypericum helps to maintain emotional balance / balanced mood / positive mood<br>**3860 - Hypericum perforatum (Common Name : St. John's Wort) - Mental health:** Contributes to emotional balance /contributes to optimal relaxation /helps to support the relaxation /helps to maintain a healthy sleep /helps maintain a positive mood<br>**4065 - Hypericum perforatum (Common Name : St. John's Wort) - Mental health:** Contributes to emotional balance and general welbeing/contributes to optimal relaxation/helps to support the relaxation and mental and physical wel being/helps to maintain a healthy sleep/helps maintain a positive mood

				**4421 - Hypericum perforatum-Herba hyperici plant-St. John Wort - Atenuates sleep disturbances due to hypericin (a napthodianthrone):** Helps in sleep disturbances.<br>**3837 - VALERIANA OFFICINALIS L. - Helps to maintain good cognitive functioning:** Contributes to recover physical and mental well-being.<br><br>**4222 - Valeriana officinalis (Common Name : Valerian) - Mental health:** Helps to maintain a natural sleep/helps maintain normal quality of sleep/helps you cope calmly with the stress of a busy lifestyle Support of mental welbeing in cases of tension and stress/contributes to optimal relaxation/helps to support the relaxation and mental and physical well being To help sleep onset, Clinically/scientifically proven to help normalise/promote sleep (onset), Valerian helps to maintain a natural sleep, To support calmness and in case of irritability Helps you cope calmly with the stress of a busy lifestyle, Support of mental well-being in cases of tension and stress, Contributes to optimal relaxation, Helps to support the relaxation and mental and physical well-being<br><br>**3925 - Lavandula angustifolia (Common Name : Lavender) - Relaxation:** Contributes to optimal relaxation /helps to support the relaxation / helps to maintain a healthy sleep/ helps in funtional abdominal complaints<br><br>**2286 - Lavandula angustifolia - common name : Lavander - Helps to find a better sleep:** / "Used to help to find a better sleep" / "Used to decrease tenseness" / "Used to decrease restleness" / "Used to decrease irritability" / "Helps to find a better sleep" / "Contributes to find a better sleep" / "Helps to decrease tenseness" / "Contributes to decrease tenseness" / "Helps to decrease restleness" / "Contributes to decrease restleness" / "Helps to derease irritability" / "Contributes to decrease irritability".<br><br>**2287 - Lavandula angustifolia - common name : Lavander - Digestive discomforts /relaxing:** used to improve the digestive comforts in case of temporary stress"<br><br>**2250 - Crataegus laevigata - common name : Hawthorn - Relaxing effect - Helps to find sleep:** / "Used to help to find a better sleep" / "Used to decrease tenseness" / "Used to decrease restleness" / "Used to decrease irritability" / "Helps to find a better sleep" / "Contributes to find a better sleep" / "Helps to decrease tenseness" / "Contributes to decrease tenseness" / "Helps to decrease restleness" / "Contributes to decrease restleness" / "Helps to derease irritability" / "Contributes to decrease irritability".
26	uva-ursi	urogenital	Contributes to the normal function of the urinary tract.	**3902 - Arctostaphylos uva ursi (Common Name: Bearberry) - Bladder health/ Health of urinary tract.** Uncomplicated infections of the lower urinary tract, such as cystitis, when antibiotic treatment is not considered essential: Support of normal bladder function
		antiseptic	Has an antiseptic effect and is used as an uroantiseptic for **the treatment** of urinary tract infections.	
		urogenital	Acts as a mild diuretic.	

35	marsh mallow, honey, propolis, primrose, mint	antimicrobial	Natural antimicrobic.	**3723 - Althaea officinalis L. (Common name: Marshmallow) - Respiratory health:** Soothing for mouth and throat / Reliefs in case of tickle in the throat and pharynx / Soothing and pleasant effect on throat, pharynx and vocal cords
		respiratory	Dilutes secretions in the respiratory tract.	**4258 - Primula veris (Common Name: Cowslip) - Health of the upper respiratory tract:** Promotes upper respiratory tract health.
		respiratory	Thanks to its unique composition, it is recommended in order to preserve the normal function of the respiratory tract.	**4259 - Primula veris L. syn. Primula officinalis L. (Common name: Cowslip) - Respiratory health:** Soothing for mouth and throat / Reliefs in case of tickle in the throat and pharynx / Soothing and pleasant effect on throat, pharynx and vocal cords
				**4468 - Primula officinalis (promrose)-radix- - it sustain the respiratory apparatus; the saponins have a secretolytic and secretomotor action**: it favours expectoration of bronchial secretions.
51	thyme, primrose, echinacea, vitamins C,E,A	respiratory	Food supplement based on thyme, primrose, echinacea and vitamins A, C, E, intended for respiratory organs and upper respiratory tract in smokers.	Vitamin A contributes to the maintenance of normal mucous membranes. Vitamin A contributes to the normal function of the immune system. Vitamin C contributes to the normal function of the immune system. Vitamin C contributes to the protection of cells from oxidative stress. Vitamin E contributes to the protection of cells from oxidative stress.
		respiratory	Vitamin A contributes to the maintenance of normal mucous membranes.	**3950 - Echinacea purpurea (Common name: Purple Coneflower Herb) - Healt of the upper respiratory tract:** adjuvant terapy of recurrent infection of the upper respiratory tract/prophylaxis of recurrent infection of the upper respiratory tract/supportive therapy for cold
		immune	Vitamin A and C contribute to the normal function of the immune system.	**4032 - Echinacea purpurea L. Moench (Common name: Echinacea) - Respiratory Health:** Soothing for mouth and throat / Reliefs in case of irritation of throat and pharynx / Soothing and pleasant effect on throat, pharynx and vocal cords
		immune	Vitamins C and E contribute to the protection of cells from oxidative stress.	**4257 - Echinacea angustifolia/pallida (Common Name : Echinacea, pale coneflower) - Immune health:** Support of the body´s defence /contributes to the /supports the immune system
		respiratory	Thyme contributes to the health and function of the respiratory tract and the protection of the mucous membranes of the tracheobronchi.	**4258 - Primula veris (Common Name: Cowslip) - Health of the upper respiratory tract:** Promotes upper respiratory tract health.
		respiratory	Primrose contributes to the health of the upper respiratory tract, calming the irritation of the throat, pharynx and vocal cords.	**4259 - Primula veris L. syn. Primula officinalis L. (Common name: Cowslip) - Respiratory health:** Soothing for mouth and throat / Reliefs in case of tickle in the throat and pharynx / Soothing and pleasant effect on throat, pharynx and vocal cords
		immune	Echinacea contributes to respiratory and immune system health.	**4468 - Primula officinalis (promrose)-radix- - it sustain the respiratory apparatus; the saponins have a secretolytic and secretomotor action**: it favours expectoration of bronchial secretions.
				**2149 - Thymus vulgaris/zygis (Common Name : Thyme) - Health of the upper respiratory tract:** Soothing for troat and chest /contributes to wellbeing of chest and throat /contributes to a fresh breath '-Good for respiratory tract and/or throat, -Soothens the respiratory tract

				**2687 - Common Thyme (Thymus vulgaris, Thymus zygis) - Supports secretion of mucus in the upper respiratory tract:** Eases expectoration. Helps with dry cough.<br>**4167 - Thymus vulgaris L. (Common name: Thyme) - Respiratory health:** Soothing for mouth and throat / Reliefs in case of irritation of throat and pharynx / Soothing and pleasant effect on throat, pharynx and vocal cords
54	N-acetylcysteine, propolis, thyme	respiratory	For dry cough, for expectoration.	**2149 - Thymus vulgaris/zygis (Common Name : Thyme) - Health of the upper respiratory tract:** Soothing for troat and chest /contributes to wellbeing of chest and throat /contributes to a fresh breath '-Good for respiratory tract and/or throat, -Soothens the respiratory tract
			It has a soothing effect on the mucous membranes of the mouth and throat.	**2687 - Common Thyme (Thymus vulgaris, Thymus zygis) - Supports secretion of mucus in the upper respiratory tract:** Eases expectoration. Helps with dry cough.
		antimicrobial	Has a potential antibacterial activity.	**4167 - Thymus vulgaris L. (Common name: Thyme) - Respiratory health:** Soothing for mouth and throat / Reliefs in case of irritation of throat and pharynx / Soothing and pleasant effect on throat, pharynx and vocal cords
		immune	Contributes to the normal function of the immune system.	**2150 - Thymus vulgaris (Common Name : Thyme) - Immune health**
56	calcium, vitamins A,B,C,D,E, carrot, coriander, watercress, mint, spinach, chamomile		Tasty food supplement for children, which provides the necessary calcium and nine vitamins.	
		multiple systems of organs	Calcium is very important for maintenance of firm bones teeth, as well as for normal muscle and nerves function.	Calcium is needed for the maintenance of normal bones. Calcium is needed for the maintenance of normal teeth. Calcium contributes to normal muscle function. Calcium contributes to normal neurotransmission.
			It also ensures the intake of vitamin D, which is needed for the absorption of calcium.	Vitamin D contributes to normal absorption/utilisation of calcium and phosphorus.
			Enriched with vitamins A,B1,B2,B3,B6,B12,C,D,E, ideal multivitamin product for for maintaining the health and vitality of children whose diet lacks some vital nutrients.	**2032 - Coriandrum sativum FRUIT - Digestion:** Helps to digest toxins. Helps maintain normal gas balance in digestive tract.
			Since all the ingredients are in liquid form, vitamins and calcium are easily absorbed.	**2033 - Coriandrum sativum FRUIT - Nervous system:** Supports nerve function.
				**2035 - Coriandrum sativum FRUIT - Cardiovascular:** Supports heart function. Helps maintain normal digestion of fats. Helps maintain normal cholesterol levels.<br>**2036 - Coriandrum sativum FRUIT - Skin:** Helps keep the skin cool.<br>**2037 - Coriandrum sativum FRUIT - Immunity & antioxidant:** Supports immune function. Has a significant antioxidant effect.<br>**2249 - Coriandrum sativum - common name: Coriander - Digestion:** / "Used to facilitate the digestion" / "Contributes to the digestive comfort" / "Helps to support the digestion" / "Contributes to support the digestion".<br>**3948 - Coriandrum sativum L. (Common name: coriander) - Appetite & digestion:** Helps to digest toxins. Helps maintain normal gas balance in digestive tract, usefull in case of dyspeptic complaints and loss of appetite.

2092 - Mentha piperita (Common Name : Mint) - Intestinal and digestive health / Stomach health: Helps to supports a healthy digestion /has a positive influence on intestinal health /contributes to digestive functions /contributes to the normal function of intestinal tract /helps keep the stomach healthy

2093 - Mentha piperita (Common Name : Mint) - Immune health

2097 - Menthae piperitae aetheroleum (Common name: Peppermint oil) - Respiratory health: Soothing for mouth and throat / Reliefs in case of irritation of throat and pharynx / Soothing and pleasant effect on throat, pharynx and vocal cords

2309 - Mentha x piperita L. (Common names: peppermint, mint) - Respiratory health: Soothing for mouth and throat / Reliefs in case of tickle in the throat and pharynx / Soothing and pleasant effect on throat, pharynx and vocal cords

2696 - Peppermint (Mentha piperita) - Carminative effect: Helps with flatulence and belly spasm.

2698 - Peppermint (Mentha piperita) - Spasmolytic effect: Helps with indigestion.

4651 - Aetheroleum menthae-Mint Volatil Oil - Digestive and intestinal health due to antimicrobial activity: Helps to support the digestion

4652 - Aetheroleum menthae-Mint Volatil Oil - Immune health due to antimicrobial activity: Volatil oil contributes to destruction of pathogen microorganisms.

4653 - Aetheroleum menthae-Mint Volatil Oil - Respiratory health due to antibacterial activity against bacteria causing respiratory tract disorders: Supports the optimal functioning of respiratory system

3312 - Chamomile (Matricaria chamomilla L.) - Respiratory health: Release of the respiratory tract - Supportive and soothing in case of dry cough, tickle in the throat - Soothing the throat - Respiratory comfort - Helps to soften respiratory troubles like coughs and sore throat in a natural way - Helps maintain respiratory health

3313 - Chamomilla recutita (L.) (Chamomile-extract) – relief of airways with common cold: relief of airways caused by common cold.

3443 - Matricaria recutita L. (Common name: Chamomile) - Respiratory health: Soothing for mouth and throat / Reliefs in case of tickle in the throat and pharynx / Soothing and pleasant effect on throat, pharynx and vocal cords

4079 - Matricaria recutita L. (Common name: Chamomile) - Respiratory health: Soothing for mouth and throat / Reliefs in case of irritation of throat and pharynx / Soothing and pleasant effect on throat, pharynx and vocal cords

2237 - Chamaemelum nobile - common name: Chamomile, Roman chamomile - Digestion: / "Used to facilitate the digestion" / "Contributes to the digestive comfort" / "Helps to support the digestion" / "Contributes to support the digestion".

				**3311 - Chamaemelum nobile - common name: Chamomile, Roman chamomile - Digestion:** "Traditionally used to facilitate the digestion" / "Used to facilitate the digestion" / "Contributes to the digestive comfort" / "Helps to support the digestion" / "Contributes to support the digestion".<br>**3928 - Matricaria recutita (Common Name : Chamomile Camomile) - Digestive health:** Helps to supports the treatment of gastro-intestinal complaints such as minor spasms epigastric distension, flatulence and belching<br>**2694 - German Chamomile (Matricaria/Chamomills recutita L.) - Spasmolytic effect:** Helps with indigestion and flatulence.<br>**4436 - Matricaria chamomilla-Flowering herb-Chamomille - Skin health:** Helps to heal the skin after burns and in case of infected wounds<br>**4437 - Matricaria recutita-flower-Chamomille - Fat metabolism:** Promotes a normal metabolism of the lipids Promotes the reduction in body weight
64	elder, rose hip, beta-glucan, rutin, vitamin C		Food supplement containing elder fruit and flower extracts, rose hip extract, beta-glucan, bioflavonoid rutin and vitamin C.	**3682 - Rosa canina (Common Name : Rose Hip) - Immune health:** Contributes to the resistance against health precarious microorganism/contributes to physical wel-being
		immune	Elder contributes to the health of the immune system. Elder flower contributes to the normal functioning of the upper respiratory tract, bronchial secretion and protection of the mucous membrane of the respiratory tract.	**3680 - Rosa canina (Common Name : Rose Hip) - Respiratory health:** helps to soothe common cold/contributes to physical wel being/contributes to the body´s defences
		respiratory<br>immune	Rose hip contributes to the function of the upper respiratory tract, bronchial secretion, protection of the mucous membrane and general well-being of the organism. Rose hip is also a natural source of vitamin C and contributes to natural immunity.	**2134 - Sambucus nigra (Common Name: Elder) - Immune health:** Support of the body´s defence /Helps to supports the immune system
			Vitamin C contributes to the normal function of the immune system, protection of cells from oxidative stress, normal energy-yielding metabolism, reduction of tiredness and fatigue, increases iron absorption.	Vitamin C contributes to the normal function of the immune system. Vitamin C contributes to the protection of cells from oxidative stress. Vitamin C contributes to normal energy-yielding metabolism. Vitamin C contributes to the reduction of tiredness and fatigue. Vitamin C increases iron absorption.
			Beta-glucan is bioactive compound with immunomodulatory property.	
68	ginkgo biloba	nervous	For improving circulation, better concentration and memory.	**2546 - Ginkgo biloba - Système nerveux Démence:** Contribue à un bon équilibre nerveux Favorise un bon équilibre mental
		CVS	Modern research has confirmed the beneficial effect of the ginkgo biloba plant extract on circulation in the whole body, especially in the brain.	**3768 - Ginkgo Biloba (Common Name : Ginkgo) - Cognitive function:** Helps the maintenace of good cognitive function /helps to maintain memory with age decline and to preserve cognitive function/enhancement of cognitive performance
		CVS	The ingredients from this plant reduce the viscosity of blood, preventing the aggregation (clogging) of platelets, and thus the formation of dangerous blood clots.	**4304 - Ginkgo Biloba (Common Name : Ginkgo) - blood system microcirculation:** for symptomatic treatment of mild to moderate cerebrovascular insufficiency

		antioxidative	Active principles (ginkgoflavonoids and terpenlactones) have a strong antioxidant effect on free radicals that damage tissues, neurons and blood vessels, increasing their firmness and elasticity.	**2261 - Ginkgo biloba [dry extract GK501 Pharmaton Dry extract from leaves of Ginkgo biloba L., drug/native extract ratio (35 - 45) : 1, solvent of extraction Acetone/Water, 6.0% terpene lactones, 24.5% ginkgoflavonoids] - For cognitive performance /blood circulation:** Help maintaining mental well-being (e.g by maintaining focus on the work memory in the short term and during moments of increased stress). Help maintaining memory with age decline and to preserve cognitive function. Help maintaining good cognitive functions, to contribute to a normal blood circulation which is associated with brain performance and reactivity. Retain and recall facts, to maintain clear thinking and to maintain day-to-day focus. Contribute to mental and cognitive activities.
		nervous	Together with the effect of better blood circulation, they lead to a reduction in the symptoms of premature dementia (forgetfulness), improve concentration, relieve migraine pain, dizziness, ringing in the ears and also **prevent** stroke by regulating cerebral circulation and reducing high blood pressure.	
77	marshmallow, primrose, chamomile, basil, vitamin C	respiratory	Helps with cough.<br><br>The product is intended for individuals with symptoms of cold and flu accompanied by cough.<br>The product combines plant extracts that, thanks to their ingredients (mucus, essential oil, saponosides), have a positive effect on the mucous membranes of the respiratory tract.	**3723 - Althaea officinalis L. (Common name: Marshmallow) - Respiratory health:** Soothing for mouth and throat / Reliefs in case of tickle in the throat and pharynx / Soothing and pleasant effect on throat, pharynx and vocal cords<br><br>**4258 - Primula veris (Common Name: Cowslip) - Health of the upper respiratory tract:** Promotes upper respiratory tract health.<br>**4259 - Primula veris L. syn. Primula officinalis L. (Common name: Cowslip) - Respiratory health:** Soothing for mouth and throat / Reliefs in case of tickle in the throat and pharynx / Soothing and pleasant effect on throat, pharynx and vocal cords
		antioxidative	Honey supports the health of the respiratory tract thanks to the presence of phytochemicals with antioxidant properties.	**4468 - Primula officinalis (promrose)-radix- - it sustain the respiratory apparatus; the saponins have a secretolytic and secretomotor action**: it favours expectoration of bronchial secretions.<br><br>**4450 - Ocimum basilicum-herb-fruits, flowers, leaf/basil - protects lung cells:** Helps to soothe common cold/pleasant for cough and croakiness/contributes to physical well-being<br>**3312 - Chamomile (Matricaria chamomilla L.) - Respiratory health:** Release of the respiratory tract - Supportive and soothing in case of dry cough, tickle in the throat - Soothing the throat - Respiratory comfort - Helps to soften respiratory troubles like coughs and sore throat in a natural way - Helps maintain respiratory health<br>**3313 - Chamomilla recutita (L.) (Chamomile-extract) - relief of airways with common cold:** relief of airways caused by common cold.<br><br>**3443 - Matricaria recutita L. (Common name: Chamomile) - Respiratory health:** Soothing for mouth and throat / Reliefs in case of tickle in the throat and pharynx / Soothing and pleasant effect on throat, pharynx and vocal cords<br><br>**4079 - Matricaria recutita L. (Common name: Chamomile) - Respiratory health:** Soothing for mouth and throat / Reliefs in case of irritation of throat and pharynx / Soothing and pleasant effect on throat, pharynx and vocal cords
78	marshmallow, honey, propolis, rose hip	antimicrobial	Natural antimicrobic.	**3680 - Rosa canina (Common Name : Rose Hip) - Respiratory health:** helps to soothe common cold/contributes to physical wel being/contributes to the body´s defences

	respiratory	Disolves secretions in the respiratory tract.<br>Thanks to its unique composition, it is recommended for maintaining the normal function of the respiratory tract.	**3723 - Althaea officinalis L. (Common name: Marshmallow) - Respiratory health:** Soothing for mouth and throat / Reliefs in case of tickle in the throat and pharynx / Soothing and pleasant effect on throat, pharynx and vocal cords

Table S6. Labels of herbal food supplements which carried claims fully non-compliant with the List of authorized health claims and ′on-hold′ claims from the EFSA Register of questions.

N°	Composition	Purpose/ effect on the organ system	Claims from the labels of herbal food supplements	′on hold′ health claims / authorised health claims
15	sunflower, immortelle, plantago, scots pine, eucalyptus, thyme	respiratory	Food supplement for children and adults with herbal extracts for the normal function of the upper respiratory tract.<br><br>Wild sunflower leaves and essential oil of silver pine needles, eucalyptus and thyme contribute to the normal function of the upper respiratory organs.	**2149 - Thymus vulgaris/zygis (Common Name : Thyme) - Health of the upper respiratory tract:** Soothing for troat and chest /contributes to wellbeing of chest and throat /contributes to a fresh breath '-Good for respiratory tract and/or throat, -Soothens the respiratory tract<br><br>**2687 - Common Thyme (Thymus vulgaris, Thymus zygis) - Supports secretion of mucus in the upper respiratory tract:** Eases expectoration. Helps with dry cough.<br><br>**4167 - Thymus vulgaris L. (Common name: Thyme) - Respiratory health:** Soothing for mouth and throat / Reliefs in case of irritation of throat and pharynx / Soothing and pleasant effect on throat, pharynx and vocal cords<br><br>**4657 - Aetheroleum thymi-Thyme Volatil Oil - Respiratory health due to antibacterial activity against bacteria causing respiratory tract disorders:** Supports the optimal functioning of respiratory system<br><br>**2763 - Pinus sylvestris - Santé de la respiration:** Facilite la respiration : Bienfaisant et adoucissant du système respiratoire Influence bénéfique sur la gorge et les voies respiratoires obstruées et aide à dégager le nez et à respirer plus librement<br><br>**4646 - Aetheroleum eucalypti-Eucalyptus Volatil Oil - Respiratory health due to antibacterial activity against bacteria causing respiratory tract disorders:** Supports the optimal functioning of respiratory system<br><br>**4036 - Eucalypti aetheroleum(Common name: Eucalyptus oil) - Respiratory Health:** Soothing for mouth and throat / Reliefs in case of irritation of throat and pharynx / Soothing and pleasant effect on throat, pharynx and vocal cords<br><br>**4262 - Eucalypti aetheroleum (Common name: Eucalyptus oil) - Respiratory Health:** adjuvant treatment of chronic obstructive respiratory complaints/adjuvant treatment bronchitis and broncal athma/ sympthomatic relief of catarrh, colds and coughs/ Soothing for mouth and throat / Reliefs in case of irritation of throat and pharynx / Soothing and pleasant effect on throat, pharynx and vocal cords<br><br>**3760 - Eucalyptus globulus La-Bill. (Common name: Eucalyptus) - Respiratory health:** Soothing for mouth and throat / Reliefs in case of tickle in the throat and pharynx / Soothing and pleasant effect on throat, pharynx and vocal cords<br>**4035 - Eucalyptus globulus La-Bill.(Common name: Eucalyptus) - Respiratory Health:** Soothing for mouth and throat / Reliefs in case of irritation of throat and pharynx / Soothing and pleasant effect on throat, pharynx and vocal cords
25	Lady's mantle	urogenital	Beneficially act against inflammation and bacteria, in polycystic ovaries, on regulation of the secretion of female hormones, bringing menstrual cycles in order.	**2203 - Alchemilla vulgaris - Menstruation:** Helps to maintain good comfort before and during menstrual cycle

		GIT / respir / CVS	Against diarrhea, respiratory tract inflammation and anemia.	**2204 - Alchemilla xanthochlora - common name: Lady's Manthe - Vascular and Vein Health:** / "Used for the good circulation of blood in microvessels" / "Helps to decrease the sensations of heavy leggs".
		musculo-skeletal	To reduce rheumatic pain, blood sugar level and elimination of water from the body.	**2714 - Alchemilla xanthochlora - common name: Lady's Manthe - Vascular and Vein Health:** "Traditionally used for the good circulation of blood in microvessels" / "Traditionally used to decrease the sensations of heavy leggs" / "Used for the good circulation of blood in microvessels" / "Helps to decrease the sensations of heavy leggs".
52	plantago, thyme, primrose, vitamin C	antimicrobial	Plantago contains tannins, pectins, citric acid, vitamin C, saponosides, a lot of mucus, oil and phytoncides, which is why it has the strongest antibacterial effect among medicinal plants.	**2149 - Thymus vulgaris/zygis (Common Name : Thyme) - Health of the upper respiratory tract:** Soothing for troat and chest /contributes to wellbeing of chest and throat /contributes to a fresh breath '-Good for respiratory tract and/or throat, -Soothens the respiratory tract
		respiratory	Its active ingredients have a beneficial effect both on the mucous membrane of the upper respiratory tract and in the digestive tract.	**2687 - Common Thyme (Thymus vulgaris, Thymus zygis) - Supports secretion of mucus in the upper respiratory tract:** Eases expectoration. Helps with dry cough.
		respiratory	The saponins contained in the primrose root help expectoration. They accelerate the secretion of mucus from the bronchi, dilute thick secretions and facilitate coughing.	**4167 - Thymus vulgaris L. (Common name: Thyme) - Respiratory health:** Soothing for mouth and throat / Reliefs in case of irritation of throat and pharynx / Soothing and pleasant effect on throat, pharynx and vocal cords
		antiseptic	Thymol from thyme and menthol have a mild antiseptic effect.	**4258 - Primula veris (Common Name: Cowslip) - Health of the upper respiratory tract:** Promotes upper respiratory tract health.
		antioxidative	The addition of vitamin C enhances **the prevention** of various infections. It increases the phagocytic power of blood cells - leukocytes, thus enhancing the body's resistance to microorganisms. It ranks among the most effective antioxidants.	**4259 - Primula veris L. syn. Primula officinalis L. (Common name: Cowslip) - Respiratory health:** Soothing for mouth and throat / Reliefs in case of tickle in the throat and pharynx / Soothing and pleasant effect on throat, pharynx and vocal cords
			Linden honey contains a number of vitamins (B1, B2, B6, E, K, C, provitamin A, etc.), plenty of enzymes, about twenty minerals, the most important of which is potassium.	**4468 - Primula officinalis (promrose)-radix- - it sustain the respiratory apparatus; the saponins have a secretolytic and secretomotor action**: it favours expectoration of bronchial secretions.
		antimicrobial	It also contains phytoncides that have bactericidal and antimycological effects.	**3791 - Plantago lanceolata L. (Common name: Ribwort plantain) - Respiratory health:** Soothing for mouth and throat / Reliefs in case of tickle in the throat and pharynx / Soothing and pleasant effect on throat, pharynx and vocal cords
		CVS / GIT / nervous	It has a beneficial effect on the normalization of blood pressure and heart function, improves anemia and disorders of the digestive and respiratory organs, relieves nervous and psychological exhaustion.	**4096 - Plantago lanceolata L. (Common name: Ribwort plantain) - Respiratory Health:** Soothing for mouth and throat / Reliefs in case of irritation of throat and pharynx / Soothing and pleasant effect on throat, pharynx and vocal cords
53	Iceland lichen, plantago, propolis, cheeses, scots pine, elder	respiratory	Food supplement for children with plant extracts, propolis and essential oil for the normal function of the mucous membrane of the upper respiratory organs.<br>Iceland lichen, cheeses leaf and elder fruit contribute to the reduction of throat irritation and the normal function of the upper respiratory tract and vocal cords.	**2134 - Sambucus nigra (Common Name: Elder) - Immune health:** Support of the body´s defence /Helps to supports the immune system<br><br>**2297 - Malva sylvestris - Pharyngeal and respiratory health**

				**2709 - Mallow (leaf and flower) Malva sylvestris - Pharyngeal health / Softening throat:** Traditionally used to soothe the throat Usually known to soothe the throat<br><br>**2847 - Malva sylvestris L. (Common name: Mallow) - -Respiratory health:** Soothing for mouth and throat / Reliefs in case of irritation of throat and pharynx / Soothing and pleasant effect on throat, pharynx and vocal cords<br><br>**3744 - Cetraria islandica (L.) Acharius s.l. (Common name: Iceland Moss) - Respiratory health:** Soothing for mouth and throat / Reliefs in case of tickle in the throat and pharynx / Soothing and pleasant effect on throat, pharynx and vocal cords<br><br>**4030 - Cetraria islandica (L.) Acharius s.l.(Common name: Iceland Moss) - Respiratory health:** Soothing for mouth and throat / Reliefs in case of irritation of throat and pharynx / Soothing and pleasant effect on throat, pharynx and vocal cords
55	iron, vitamins B1,B2,B6,B12,C, carrot, nettle, spinach, common couch, fennel, brown algae, hibiscus	multiple systems of organs	It provides iron with high absorption capacity in combination with B group vitamins, carefully selected plant extracts and vitamin C, which increase its absorption.	**2152 - Urtica dioica (Common Name : Nettle) - Invigoration of the body:** Support the body´s vitality /helps to make you feel more energetic
			It is very well tolerated and does not cause digestive disturbances.	**2692 - Fennel (Foeniculum vulgare) - Spasmolytic and carminative effect:** Helps with flatulence and belly spasm.
			It helps to maintain optimal iron levels in the blood, preserve energy and physical condition, as well as overall good health and vitality.	**4648 - Aetheroleum foeniculi-Fennel Volatil Oil - Respiratory health due to antibacterial activity against bacteria causing respiratory tract disorders:** Volatil oil contributes to destruction of pathogen microorganisms.
			For the protection of the whole family's health!	**4649 - Aetheroleum foeniculi-Fennel Volatil Oil - Digestive health due to antimicrobial activity:** Helps to support the digestion
			In the case of iron deficiency caused by insufficient intake of iron from food or increased loss, such as more abundant menstrual bleeding.	**2051 - Foeniculum vulgare ssp. Cappillaceum var. vulgare. DRIED FRUIT - Appetite, digestion & elimination:** Supports appetite, digestion and elimination. Supports the health of the digestive tract.
			It provides sufficient iron during periods of increased need, such as during pregnancy and breastfeeding or for the healthy growth and development of children.	**2052 - Foeniculum vulgare ssp. Cappillaceum var. vulgare. DRIED FRUIT - Immunity. Antioxidant:** Supports the immune system. Antioxidant activity
			During recovery period from **illness**. For athletes, to replenish iron stores in the blood and muscles.	**2053 - Foeniculum vulgare ssp. Cappillaceum var. vulgare. DRIED FRUIT - Postpartum:** Supports production of breast milk. Helps maintain breast health during breast feeding.
			Tasty food supplement based on the physiological properties of selected vitamins, iron, as well as physiological properties of active principles of selected herbal water extracts and concentrates of selected fruits.	**2054 - Foeniculum vulgare ssp. Cappillaceum var. vulgare. DRIED FRUIT - Menses:** Helps maintain comfortable menstrual cycle.
			Iron is an essential dietary ingredient that enables red blood cells to transport oxygen to all parts of the body.	**2055 - Foeniculum vulgare Mill. (Common name: Fennel) - Respiratory health:** Soothing for mouth and throat / Reliefs in case of irritation of throat and pharynx / Soothing and pleasant effect on throat, pharynx and vocal cords

			It contains highly resorbable iron in the form of a food supplement based on yeast extract. Fruit acids and herbal extracts have mild digestive effects and prevent digestive problems, which are common in iron supplements. It contains nutrients necessary for growth and maintenance of physical condition and health. It alleviates persistent feeling of fatigue.<br><br>It is particularly suitable for women (including pregnant and breastfeeding women), but also for men, growing children, and individuals whose diet is low in natural iron and vitamins.	**2056 - Foeniculi aetheroleum (Common name: Fennel oil) - -Respiratory Health:** Soothing for mouth and throat / Reliefs in case of irritation of throat and pharynx / Soothing and pleasant effect on throat, pharynx and vocal cords<br><br>**3392 - Hibiscus (Hibiscus sabdariffa L.) - Tonus/ Vitality:** Helps in case of fatigue - Helps to make you feel more energetic - Enhancement of vitality/energy - Helps to support body's vitality<br><br>**2268 - Hibiscus sabdariffa - common name : Hibiscus - Invigoration of body:** / "Used to feel more energetic" / "Helps to find more energy" / "Contributes to find more energy" / "Used for mental and physical fatigue" / "Helps to enhance mental and physical capacities" / "Contributes to enhance mental and physical capacities" / "Helps to strengthen the body" / "Contributes to strengthen the body" / "Supports energetic alertness" / "Tonic effect" / "Makes you feel more energetic" / "Has stimulating and tonic properties that contribute to the resistance against mental and physical fatigue"<br><br>**2269 - Hibiscus sabdariffa - common name : Hibiscus - Vascular and vein health:** / "Used for the good circulation of blood in microvessels" / "Helps to decrease the sensations of heavy leggs"
61	linden, lemon balm, chamomile, passion flowers, sweet orange, lavender	nervous	It helps establish physiological sleep patterns in children during the night.	**2286 - Lavandula angustifolia - common name : Lavander - Helps to find a better sleep:** / "Used to help to find a better sleep" / "Used to decrease tenseness" / "Used to decrease restleness" / "Used to decrease irritability" / "Helps to find a better sleep" / "Contributes to find a better sleep" / "Helps to decrease tenseness" / "Contributes to decrease tenseness" / "Helps to decrease restleness" / "Contributes to decrease restleness" / "Helps to derease irritability" / "Contributes to decrease irritability".<br><br>**2287 - Lavandula angustifolia - common name : Lavander - Digestive discomforts /relaxing:** used to improve the digestive comforts in case of temporary stress"<br><br>**2302 - Melissa officinalis - common name : melissa, lemon balm, balm mint, sweet balm, common balm - Relaxing effect - helps to find sleep:** "Used to help to find a better sleep" / "Used to decrease tenseness" / "Used to decrease restleness" / "Used to decrease irritability" / "Helps to find a better sleep" / "Contributes to find a better sleep" / "Helps to decrease tenseness" / "Contributes to decrease tenseness" / "Helps to decrease restleness" / "Contributes to decrease restleness" / "Helps to derease irritability" / "Contributes to decrease irritability".<br><br>**2564 - Passiflore plante - Système nerveux Favorise le sommeil:** A utiliser pour un sommeil sain Favorise un repos nocturne bon et sain<br><br>**2636 - extract of Passion flower (Passiflora incarnata) - herbal sedative:** helps to induce calm rest and sleep helps to calm down after the argument and excitedness recommended to people feeling week and fatigued helps to induce relaxation

				**3616 - Tilia platyphyllos - common name : Linden - Relaxing effect - Helps to find sleep:** "Traditionally used to help to find a better sleep" / "Traditionally used to decrease tenseness" / "Traditionally used to decrease restleness" / "Traditionally used to decrease irritability" / "Used to help to find a better sleep" / "Used to decrease tenseness" / "Used to decrease restleness" / "Used to decrease irritability" / "Helps to find a better sleep" / "Contributes to find a better sleep" / "Helps to decrease tenseness" / "Contributes to decrease tenseness" / "Helps to decrease restleness" / "Contributes to decrease restleness" / "Helps to derease irritability" / "Contributes to decrease irritability".
69	propolis, plantago lanecolata, vitamin C	antibiotic	Propolis is the most important bee product and natural antibiotic.	No defined "on hold" claims for propolis
			The main ingredients are various plant resins and wax that bees process with the secretion of their glands.	**3510 - PLANTAGO LANCEOLATA L. - Contributes to maintain a normal intestinal function:** Stimulates the growth of beneficial intestinal micro flora.
		antioxidative	It contains a wide spectrum of physiologically active substances such as bioflavonoids, polyphenols, anthocyanins, phytohormones, essential oils, pollen, vitamins, micro and macro elements, and therefore represents an important food supplement and a powerful protector.	**3512 - PLANTAGO LANCEOLATA L. - Helps to maintain a healthy intestinal microbial balance:** Stimulates the growth of beneficial intestinal micro flora.
		antimicrobial	It exhibits antibacterial, antifungal, antiviral, antiparasitic, antioxidant and immunostimulating effects.	**3513 - PLANTAGO LANCEOLATA L. - Hepatoprotective:** Helps to maintain a healthy liver function, supporting the digestion and the body purification.
		multiple systems of organs	It has a beneficial effect on strengthening the body's resistance, and can be used for inflammation of the mouth, throat, nose and ear, as well as in gynecology and dermatocosmetology, for superficial wounds and infections on the skin and mucous membranes, activating the enzymes of cellular metabolism, thereby stimulating the processes of regeneration and epithelization of damaged tissue.	**3791 - Plantago lanceolata L. (Common name: Ribwort plantain) - Respiratory health:** Soothing for mouth and throat / Reliefs in case of tickle in the throat and pharynx / Soothing and pleasant effect on throat, pharynx and vocal cords
		immune	Together with active principles from plantago and vitamin C, it represents an original food supplement and a strong protective factor for the body in the fight against various infections.	**4096 - Plantago lanceolata L. (Common name: Ribwort plantain) - Respiratory Health:** Soothing for mouth and throat / Reliefs in case of irritation of throat and pharynx / Soothing and pleasant effect on throat, pharynx and vocal cords
83	wild garlic	CVS	The active ingredients of wild garlic leaf have a beneficial effect on complaints caused by blood circulation disorders, so wild garlic is used as an aid in alleviating the symptoms of initial or advanced arteriosclerosis caused by aging, as well as in individuals with high blood pressure.	**2174 - Allium ursinum (Common Name : Bear's garlic) - Heart health / Vascular system:** Helps to maintain the healthy functioning of heart and blood vessels
		immune	Due to the beneficial effect of its ingredients on the immune system, the use of wild garlic is recommended in order to reduce the risk of infections and as a mean of blood purification.	**3210 - allium ursinum - drainant des métaux lourds, métabolisme du cholestérol, fonction circulatoire, fonction intestinale:** régule le métabolisme du cholestérol, régule l'activité cardiaque,
		GIT	It has a beneficial effect on conditions that require the regulation of digestion, it helps to alleviate gastric	

			and intestinal parasites in children.	
85	garlic	CVS<br>CVS<br>antiseptic / antimicrobial	Garlic tincture inhibits platelet aggregation, improves blood circulation, lowers blood pressure and has a beneficial effect on various parameters in fat metabolism.<br>Using this tincture improves the symptoms of initial or advanced arteriosclerosis.<br>Additionally, the preparation exhibits antiseptic, antifungal and digestive effects.	**3209 - Allium sativum (Garlic) - Antibacterial, antioxidative activity, strengthens immune system.**<br>**1993 - Allium sativum (aged garlic) (Common Name : Aged garlic) - Stress:** Contributes to the resistance against temporary stress<br>**2208 - Allium sativum - common name : Garlic - Vascular and Vein Health:** / "Used for the good circulation of blood in microvessels" / "Helps to decrease the sensations of heavy leggs"<br>**2361 - Garlic bulb (Allium sativum bulbus); - Respiratory health:** Garlic helps maintain the healthy function of the respiratory system.<br>**1994 - Allium sativum (aged garlic) (Common Name : Aged garlic) - Liver health:** Helps to maintain a healthy liver function

Table S7. Labels of herbal food supplements which carried health claims for ingredients for which there were no defined ′on-hold′ claims in the EFSA Register of questions for the specified purposes.

N°	Composition	Purpose/effect on the organ system	Claims from the labels of herbal food supplements	′on hold′ health claims / authorised health claims
20	aronia		Aronia fruit contains vitamins C, A, E, P, and B complex, as well as a large number of minerals.	No defined ′on hold′ claims
		immune / CVS	It helps to strengthen the immune system, stimulates circulation and has a beneficial effect on reducing cholesterol and glucose in the blood.	
48	common ivy	respiratory	Helps with all types of cough.	No defined ′on hold′ claims
58	propolis	immune	Propolis contains a wide spectrum of physiologically active substances such as: bioflavonoids, phytohormones, essential oils, pollen, vitamins, microelements and macroelements.	No defined ′on hold′ claims
		antimicrobial / antitumor	Propolis has bacteriostatic, bactericidal, antiviral, antifungal, immunostimulatory and antitumor properties.	
59	propolis	immune	The product is intended to strengthen the body.	No defined ′on hold′ claims
66	speedwell, dandelion, woundwort, birch, field horsetail	urogenital	Food supplement based on a mixture of herbal ingredients that contributes to preserving the normal functioning of the organs of the urinary tract.	No defined ′on hold′ claims
			The product is not intended for the diagnosis, treatment or cure of any disease.	
70	propolis	antibiotic	Propolis is the most important bee product and natural antibiotic.	No defined ′on hold′ claims
			The main ingredients are various plant resins and wax that bees process with the secretion of their glands.	
			It contains a wide spectrum of physiologically active substances such as bioflavonoids, polyphenols, anthocyanins, phytohormones, essential oils, pollen, vitamins, micro and macro elements, and therefore represents an important food supplement and a powerful protector.	
		antimicrobial	It exhibits antibacterial, antifungal, antiviral, antiparasitic, antioxidant and immunostimulating effects.	
		multiple systems of organs	It has a beneficial effect on strengthening the body's resistance, and can be used for inflammation of the mouth, throat, nose and ear, as well as in gynecology and dermatocosmetology, for superficial wounds and infections on the skin and mucous membranes, activating the enzymes of cellular metabolism, thereby stimulating the processes of regeneration and epithelization of damaged tissue.	
72	propolis	immune	The product is intended to strengthen resistance and general body condition in children over 6 years old.	No defined ′on hold′ claims
			The product is not a medicine nor a diagnostic device.	
74	propolis, royal jelly	immune	The product is intended to strengthen resistance and general body condition.	No defined ′on hold′ claims
			The product is not a medicine nor a diagnostic device.	

Table S8. Precautionary and warning statements on the supplement's usage listed on the labels of herbal food supplements.

N°	Cannot be used as a substitute for a varied diet and a healthy lifestyle	Recommended daily doses cannot be exceeded	Usage not recommended in...	Consult a doctor...	Interactions	Limitations in use in certain conditions/diseases	Side effects	Alergic reactions
1	+	+	sensitive individuals, young children, pregnant and lactating women		with antitussives		disorders in the functioning of the GIT (gastric disorders, nausea, vomiting), hypersensitivity reactions	hypersensitivity reactions
2	+	+	sensitive individuals, young children, pregnant and lactating women		with antitussives, with other medicines 1 hour apart		disorders in the functioning of the GIT (gastric disorders, nausea, vomiting), hypersensitivity reactions	in hypersensitive individuals, the development of symptoms of allergic reactions is possible
3	+	+	sensitive individuals, children under the age of 3, pregnant and lactating women					
4	+	+	sensitive individuals, children under the age of 4, pregnant and lactating women		with antitussives			
5	+	+	sensitive individuals, children under the age of 4, pregnant and lactating women		with antitussives		disorders in the functioning of the GIT (nausea, vomiting, diarrhea)	in hypersensitive individuals, the development of symptoms of allergic reactions is possible (hives, difficulty breathing)
6	+	+	sensitive individuals, children under the age of 4, pregnant and lactating women			history of acute obstructive laryngitis, asthma, diabetes, caution in individuals with gastritis, gastric ulcer	nausea and other GIT disorders	

7	+	+	sensitive individuals, children under the age of 3, pregnant and lactating women			diabetes		
8	+	+	allergic individuals, children under the age of 3	pregnant and lactating women	contains marshmallow root which may reduce the absorption of simultaneously administered medication, so it should be taken one hour before or after taking the medication	children suffering from asthma		
9	+	+	sensitive individuals, pregnant and lactating women	for children under the age of 12 consult a pediatrician		caution in diabetes		
10	+	+	sensitive individuals, pregnant and lactating women	for children under the age of 12 consult a pediatrician		caution in diabetes	possible milder GIT complaints	
11	+	+	children under the age of 4					
12	+	+	sensitive individuals, pregnant and lactating women, children under the age of 3		it is necessary to make a break of one hour between taking the product and other medicines	contains sugar, caution in diabetes		in hypersensitive individuals, the development of symptoms of allergic reactions is possible (dermatitis)
13	+	+	sensitive individuals, pregnant and lactating women, children under the age of 4			asthma sufferers and children with a history of obstructive laryngitis; caution in gastritis and gastric ulcer; contains sucrose (sugar), caution in individuals with diabetes	hypersensitivity reactions, mild and transient digestive disorders, appearance of a laxative effect	hypersensitivity reactions
14	+	+	sensitive individuals	pregnant and lactating women				

15	+	+	sensitive individuals	pregnant and lactating women		individuals with bronchial asthma and severe cough, biliary obstruction, heart failure and circulatory disorders, inflammatory diseases of the digestive tract or gall bladder, severe liver diseases		
16	+	+	sensitive individuals					
17	+	+	sensitive individuals, pregnant and lactating women, children under the age of 3		when taken with oral antibiotic therapy, it should be taken 2 hours after the administered dose of the antibiotic	immunocompromised individuals, individuals on immunosuppressive therapy including allograft patients, after surgical operations, individuals with bloody diarrhea, central venous catheters		
18	+	+	sensitive individuals, children under the age of 1	pregnant and lactating women		contains sugar, caution in diabetes		
19	+	+	sensitive individuals, pregnant and lactating women, children under the age of 1		products based on astragalus are not recommended for individuals on immunosuppressive therapy, corticosteroids and oral anticoagulants	contains sugar, caution in diabetes		
20	+		children, pregnant and lactating women, sensitive individuals	consult a doctor / pharmacist before use				
21	+		children, pregnant and lactating women, sensitive individuals	consult a doctor / pharmacist before use				
22	+		children, pregnant and lactating women, sensitive individuals	consult a doctor / pharmacist before use				
23	+		children, pregnant and lactating women, sensitive individuals	consult a doctor / pharmacist before use				

24	+		children, pregnant and lactating women, sensitive individuals	consult a doctor / pharmacist before use				
25	+		children, pregnant and lactating women, sensitive individuals	consult a doctor / pharmacist before use				
26	+		children, pregnant and lactating women, sensitive individuals	consult a doctor / pharmacist before use				
27			do not use in pregnant women during the last 3 months of pregnancy	consult a doctor / pharmacist about indications, precautions and side effects			in case of eye irritation, rinse with cold water as soon as possible and, if needed, seek medical help	
28	+	+	children, pregnant and lactating women, sensitive or allergic individuals			individuals with asthma		
29	+	+	sensitive individuals, pregnant and lactating women, children under the age of 6	consult a doctor / pharmacist before use				
30	+	+	children under the age of 4, sensitive individuals, pregnant and lactating women	individuals with GIT problems and diabetics should consult a doctor before use			color E 110 can adversely affect activity and attention in children	
31	+	+	sensitive individuals, pregnant and lactating women		it should not be administered simultaneously with medications, but rather an hour before or after taking any medication	caution in patients with gastric inflammation (gastritis) or gastric ulcer		
32	+		sensitive individuals, children under the age of 3			the product is not suitable for use by diabetics		
33	+	+	sensitive individuals, pregnant and lactating women					

34	+	+	sensitive individuals, pregnant and lactating women		it should not be administered simultaneously with medications, but rather an hour before or after taking any medication			
35	+	+	sensitive individuals, children under the age of 6, pregnant and lactating women		oral antibiotics should be taken at least 2 hours before using the product; if administered simultaneously with nitroglycerin, headache may occur	individuals suffering from asthma, acute obstructive laryngitis, gastritis, stones in the gall bladder and bile ducts		
36	+	+	children under the age of 3	consult a doctor / pharmacist before use				
37	+	+	sensitive individuals, children under the age of 4, pregnant and lactating women			children with a history of acute obstructive laryngitis, children suffering from asthma; caution in diabetics; caution in children with gastritis, gastric ulcer	GIT problems (nausea, vomiting, gastric problems) and allergic reactions; excessive use of primrose root products can lead to stomach disorders, vomiting and diarrhea	allergic reactions
38	+	+	sensitive individuals	consult a doctor / pharmacist before use				
39	+	+	sensitive individuals					
40	+	+	sensitive individuals	pregnant and lactating women				
41	+	+	sensitive individuals	pregnant and lactating women		gastric and duodenal ulcer		
42	+	+	sensitive individuals, pregnant and lactating women, under the age of 18	individuals suffering from other diseases or using medications are advised to consult a doctor before use	do not use together with anticoagulants	do not use in case of liver disease	GIT disturbances and allergic reactions	allergic reactions
43	+	+	sensitive individuals, pregnant and lactating women, under the age of 18	individuals suffering from other diseases or using medications		women with pituitary tumor and breast cancer, on hormone therapy, those taking oral		

				are advised to consult a doctor before use		contraceptives		
44	+	+	sensitive individuals, pregnant and lactating women, under the age of 12	individuals suffering from other diseases or using medications are advised to consult a doctor before use				
45	+	+	sensitive individuals, pregnant and lactating women, under the age of 6	individuals suffering from other diseases or using medications are advised to consult a doctor before use				
46	+	+	sensitive individuals, pregnant and lactating women, under the age of 12		the use of echinacea-based products is contraindicated in individuals suffering from leukemia, with progressive systemic diseases (multiple sclerosis, lupus erythematosus, tuberculosis, etc.), with AIDS, HIV infections, autoimmune diseases, in individuals on immunosuppressive therapy	leukemia, progressive systemic diseases (multiple sclerosis, lupus erythematosus, tuberculosis, etc.), AIDS, HIV infections, autoimmune diseases, individuals on immunosuppressive therapy		
47	+	+	sensitive individuals, children under the age of 3, pregnant and lactating women		in the case of simultaneous administration with other medications, leave a gap of at least 1 hour between taking the food supplement and the medication; simultaneous use with antitussives is not recommended			in hypersensitive individuals, the development of symptoms of allergic reactions is possible

48	+	+	sensitive individuals, children under the age of 3, pregnant and lactating women		simultaneous use with antitussives is not recommended	caution in individuals with gastritis or gastric ulcer	disturbances in GIT functioning may occur (nausea, vomiting, diarrhea)	in hypersensitive individuals, the development of symptoms of allergic reactions is possible (hives, difficulty breathing)
49	+	+	sensitive individuals, pregnant and lactating women, children under the age of 3	the product should be used under medical supervision	products with pelargonium (geranium) are not recommended for individuals on anticoagulant therapy and people planning surgical intervention; caution in individuals with liver disease, psoriasis, rheumatoid arthritis, lupus, and autoimmune diseases due to its ability to activate antibodies and cause autoimmune disorders	contains sugar, caution in diabetes		
50	+	+	sensitive individuals, pregnant and lactating women			does not contain sugar, can be used by diabetics		
51	+	+	sensitive, alergic individuals, pregnant and lactating women, under the age of 18			caution in diabetes; not recommended in individuals with tuberculosis, leukosis, collagenosis, multiple sclerosis, AIDS, other autoimmune diseases	prolonged use of echinacea, longer than 2 months, can lead to the opposite effect - suppression of the immune system	
52	+	+	sensitive individuals, pregnant and lactating women	consult a doctor; for children under the age of 12 consult a pediatrician		caution in diabetes	possible milder GIT complaints	
53	+	+	sensitive individuals	pregnant and lactating women				

54	+	+	sensitive individuals, pregnant and lactating women		simultaneous use with antitussives is not recommended	chronic liver diseases, kidney stones, gastric ulcers	allergic reactions, GIT disorders; while using preparations based on N-acetylcysteine, it is necessary to increase fluid intake	allergic reactions
55	+	+						
56	+	+						
57	+	+	pregnant and lactating women, children under the age of 3, sensitive individuals	for use in children under the age of 6, it is necessary to consult a doctor, pediatrician		liver diseases, increased risk of bleeding		
88			sensitive and alergic individuals					
99	+	+	allergic individuals, children under the age of 6, pregnant and lactating women					
60	+	+	sensitive individuals, children under the age of 3					
61	+	+	sensitive individuals, children under the age of 3, pregnant and lactating women					
62	+	+	sensitive individuals, children under the age of 6, pregnant and lactating women	before use in children older than 6 years, parents should consult a doctor; individuals diagnosed with a medical condition and/or taking medication				
63	+	+						
64	+	+	sensitive individuals, children under the age of 3, pregnant and lactating women			caution in diabetes		

65	+	+		young children age 1 to 3 years only upon pediatrician's advice; children and adolescents up to 14 years, pregnant and lactating women, only upon doctor's or pharmacist's advice		suitable for diabetics		
66	+	+	children under the age of 16, pregnant and lactating women, sensitive individuals			stomach ulcer and gastritis, biliary obstruction, ileus, edema caused by kidney dysfunction		
67	+	+	sensitive individuals, pregnant and lactating women, under the age of 18					
68	+	+	sensitive individuals, pregnant and lactating women, under the age of 18	consult a doctor	caution in individuals on anticoagulant therapy (there is a possibility of bleeding thus stop using these drops before surgical intervention), anticonvulsant therapy	caution in diabetes, hypertensive patients, depressive states	headache, functional GIT disorders, allergic reactions	allergic reactions
69			sensitive individuals, pregnant and lactating women, under the age of 12					
70			sensitive individuals, pregnant and lactating women, under the age of 12					
71	+	+	alergic individuals, pregnant and lactating women, under the age of 6				hypersensitivity reactions, insomnia, GIT complaints	hypersensitivity reactions
72	+	+	alergic individuals, pregnant and lactating women, under the age of 6					

73	+	+	alergic individuals, pregnant and lactating women, under the age of 6					
74	+	+	alergic individuals, pregnant and lactating women, under the age of 6		possible interaction of royal jelly with warfarin	asthma, tendency to allergies	royal jelly can worsen the condition in people with dermatitis	
75	+	+	sensitive individuals, young children, pregnant and lactating women		in the case of simultaneous administration with other medications, leave a gap of at least 1 hour between taking the food supplement and the medication; simultaneous use with antitussives is not recommended			in hypersensitive individuals, the development of symptoms of allergic reactions is possible
76	+	+	sensitive individuals, pregnant and lactating women, children under the age of 3		preparations of marshmallow root may reduce the absorption of simultaneously administered medication, so they should be taken one hour before or two hours after taking the medication	not suitable for diabetics due to high sugar content		
77	+	+	allergic individuals, children under the age of 3	pregnant and lactating women	contains marshmallow root which may reduce the absorption of simultaneously administered medication, so it should be taken one hour before or after taking the medication	children suffering from asthma, caution in diabetics, individuals with gastritis, gastric ulcer		
78	+	+	sensitive individuals, pregnant and lactating women, children under the age of 3		oral antibiotics should be taken at least 2 hours before using the product; if administered simultaneously with nitroglycerin, headache may occur	asthma, acute obstructive laryngitis, gastritis, stones in the gall bladder and bile ducts		

79	+	+	sensitive individuals, pregnant and lactating women, children under the age of 3		contains marshmallow root which may reduce the absorption of simultaneously administered medication, so it should be taken one hour before or after taking the medication	children with a history of acute obstructive laryngitis, children suffering from asthma, caution in diabetics, individuals with gastritis, gastric ulcer	nausea and other GIT disorders	
80	+	+	sensitive individuals, pregnant and lactating women, children under the age of 3					
81	+	+	sensitive individuals, pregnant and lactating women, persons under the age of 18	individuals taking medicines should consult a doctor before use	the use of hawthorn drops is contraindicated in individuals on therapy with cardiotonic heterosides and drugs from the group of beta-blockers and antiarrhythmics			
82	+	+	sensitive individuals, pregnant and lactating women, children under the age of 12					
83								
84	+	+	sensitive individuals, pregnant and lactating women, children under the age of 12		the product should not be taken with sedative drugs		valerian can affect the ability to drive a vehicle or operate machinery, thus the product should not be taken 2-3 hours before	
85								
86	+	+	allergic and sensitive individuals, children under the age of 3					
87	+	+	pregnant and lactating women, allergic and sensitive individuals		if you are taking anticoagulants or being treated for diabetes, ask your doctor about using this product	if you are taking anticoagulants or being treated for diabetes, ask your doctor about using this product		
